# Enhanced stability of hippocampal place representation caused by reduced magnesium block of NMDA receptors in the dentate gyrus

**DOI:** 10.1186/1756-6606-7-44

**Published:** 2014-06-04

**Authors:** Yuichiro Hayashi, Yoko Nabeshima, Katsunori Kobayashi, Tsuyoshi Miyakawa, Koichi Tanda, Keizo Takao, Hidenori Suzuki, Eisaku Esumi, Shigeru Noguchi, Yukiko Matsuda, Toshikuni Sasaoka, Tetsuo Noda, Jun-ichi Miyazaki, Masayoshi Mishina, Kazuo Funabiki, Yo-ichi Nabeshima

**Affiliations:** 1 Horizontal Medical Research Organization, Kyoto University Graduate School of Medicine, Kyoto 606-8501, Japan; 2 Medical Innovation Center, Kyoto University Graduate School of Medicine, Kyoto 606-8501, Japan; 3 Department of Pathology and Tumor Biology, Kyoto University Graduate School of Medicine, Kyoto 606-8501, Japan; 4 Department of Pharmacology, Graduate School of Medicine, Nippon Medical School, Bunkyo-ku, Tokyo 113-8602, Japan; 5 Genetic Engineering and Functional Genomics, Frontier Technology Center, Kyoto University Graduate School of Medicine, Kyoto 606-8501, Japan; 6 Division of Systems Medical Science, Institute for Comprehensive Medical Science, Fujita Health University, Toyoake, Aichi 470-1192, Japan; 7 National Institute for Physiological Sciences, Center for Genetic Analysis of Behavior, 38 Nishigonaka Myodaiji, Okazaki, Aichi 444-8585, Japan; 8 National Institute of Neuroscience, National Center of Neurology and Psychiatry, Kodaira, Tokyo 187-8502, Japan; 9 Meiji Institute of Research and Development, Meiji Milk Products Company Limited, Tokyo 136-8908, Japan; 10 Laboratory of Integrative Bioscience, Graduate School of Biomedical Sciences, Hiroshima University, 1-2-3 Kasumi, Minami, Hiroshima 734-8553, Japan; 11 Department of Cell Biology, Japanese Foundation for Cancer Research, Cancer Institute, Tokyo 135-8550, Japan; 12 Department of Nutrition and Physiological Chemistry, Osaka University Medical School, Osaka 565-0871, Japan; 13 Department of Molecular Neurobiology and Pharmacology, Graduate School of Medicine, University of Tokyo, Tokyo 113-0033, Japan; 14 Osaka Bioscience Institute, 6-2-4 Furuedai, Suita, Osaka 565-0874, Japan; 15 Core Research for Evolutional Science and Technology (CREST), Kawaguchi 332-0012, Japan; 16 Institute of Biomedical Research and Innovation, Foundation of Biomedical Research and Innovation, 2-2 Minatojima-Minamimachi, Chuo-ku, Kobe 650-0047, Japan

**Keywords:** Mg^2+^ block, NMDA receptor, Dentate gyrus, Place cell

## Abstract

**Background:**

Voltage-dependent block of the NMDA receptor by Mg^2+^ is thought to be central to the unique involvement of this receptor in higher brain functions. However, the *in vivo* role of the Mg^2+^ block in the mammalian brain has not yet been investigated, because brain-wide loss of the Mg^2+^ block causes perinatal lethality. In this study, we used a brain-region specific knock-in mouse expressing an NMDA receptor that is defective for the Mg^2+^ block in order to test its role in neural information processing.

**Results:**

We devised a method to induce a single amino acid substitution (N595Q) in the GluN2A subunit of the NMDA receptor, specifically in the hippocampal dentate gyrus in mice. This mutation reduced the Mg^2+^ block at the medial perforant path–granule cell synapse and facilitated synaptic potentiation induced by high-frequency stimulation. The mutants had more stable hippocampal place fields in the CA1 than the controls did, and place representation showed lower sensitivity to visual differences. In addition, behavioral tests revealed that the mutants had a spatial working memory deficit.

**Conclusions:**

These results suggest that the Mg^2+^ block in the dentate gyrus regulates hippocampal spatial information processing by attenuating activity-dependent synaptic potentiation in the dentate gyrus.

## Background

NMDA receptors (NMDARs) contribute to the majority of excitatory synaptic transmission in the brain, and are key molecules involved in synaptic plasticity, learning, and memory [1]. The activation of NMDAR channels is linked to the membrane potential by the voltage-dependent Mg^2+^ block [2,3], allowing these channels to sense the coincidence of pre- and postsynaptic activity [4]. The use of pharmacological blockades and transgenic animals has shown that the NMDARs play a crucial role in various types of learning [5]. Recently, it has been demonstrated that elevated brain magnesium concentrations enhance memory function [6], suggesting that Mg^2+^ block of NMDARs is involved in memory. Furthermore, *Drosophila* overexpressing an NMDAR defective for Mg^2+^ block has defects in long-term memory [7]. However, the *in vivo* role of the Mg^2+^ block in vertebrates still remains unclear. NMDARs defective for the Mg^2+^ block cause perinatal lethality in mice expressing these receptors [8].

A single amino acid substitution is known to greatly change the Mg^2+^ blockade of the NMDAR. Functional NMDARs are likely hetero-oligomers comprising two types of subunits, GluN1 and GluN2. Each subunit has four predicted membrane-associated segments (M1–M4). A single asparagine residue in M2 is critical for voltage-dependent Mg^2+^ blockade. Replacement of asparagine 598 of the GluN1 subunit with glutamine strongly reduces the sensitivity of the NMDAR channel to Mg^2+^ block [9,10]. A similar mutation in the GluN2 subunits (N595Q) also strongly reduces the block by Mg^2+^[9]. In the present study, we developed a new method for introducing a desired mutation into the gene of interest in a spatially restricted manner. Using this technique, we selectively introduced a single amino acid mutation (N595Q) into the GluN2A subunit in the dentate gyrus (DG) and tested the involvement of the Mg^2+^ block of NMDARs in hippocampal computational function.

The hippocampus is one of the most widely studied brain regions because of its simple structure and central role in memory formation in both humans and other mammals [11,12]. Anatomically, the hippocampus can be divided into three structurally dissimilar areas: the DG, CA3, and CA1, and the DG is the first region involved in the hippocampal trisynaptic circuit [13]. We used hippocampal place cell activity to test the role of the DG Mg^2+^ block in hippocampal information processing. Place cells are neurons that exhibit a high firing rate when an animal is in a specific location in an environment [14,15]. The properties of place cells have been used to gain insights into neural computation in the hippocampus [16-18].

Here we found that expression of receptors with the N595Q substitution in GluN2A in the granule cells of the dentate gyrus reduced the Mg^2+^ block of NMDAR-mediated synaptic currents and facilitated activity-dependent synaptic potentiation at medial perforant path–granule cell synapses. Hippocampal place representation in the mutants was more stable than that of the controls and place representation showed low sensitivity to visual differences. These results imply that enhanced synaptic potentiation resulting from the decrease in Mg^2+^ blocking stabilizes place representation but impairs pattern separation. The mutants also showed deficits in working memory, indicating that the Mg^2+^ block contributes to spatial learning.

## Results

### Generation of the GluN2A (N595Q) mouse

**Figure 1:**
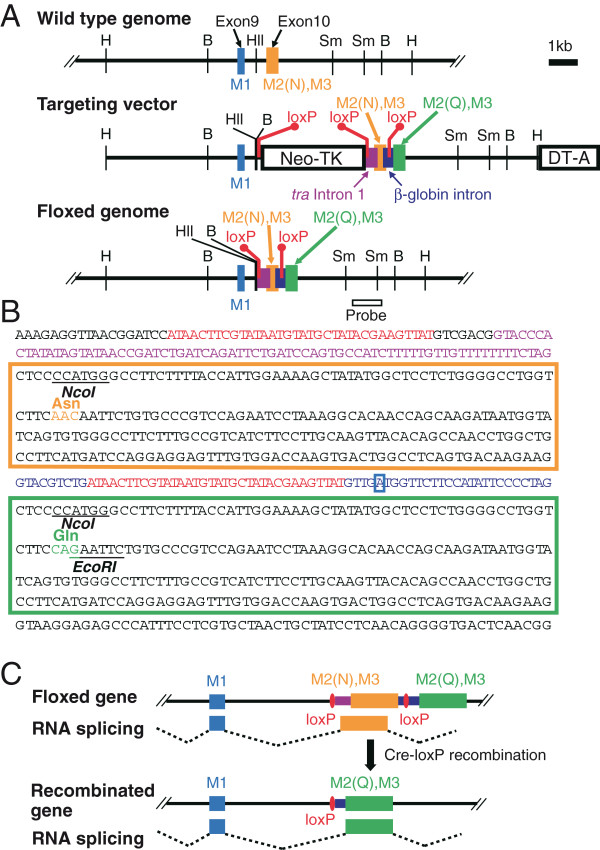
**Targeted manipulation of the *GluN2A *gene. (A)** Top: the wild-type *GluN2A* gene locus, Middle: the targeting vector containing the WT and mutant exon 10, the artificial introns, and three loxP sequences. Bottom: the floxed *GluN2A* gene. The open horizontal bar represents the probe used for Southern blot analyses. Relevant restriction sites are indicated: B, *Bam*HI; H, *Hin*dIII; HII, *Hin*cII; Sm, *Sma*I. **(B)** The artificial exon–intron sequences are described. The loxP sequences are shown in red. WT exon 10 and mutant exon 10 are enclosed in orange and green boxes, respectively. The fruit fly *tra* gene intron 1 sequences (including Sxl-binding splicing acceptor) and the splice donor and acceptor sites of the mouse β-globin intron are shown in violet and blue, respectively. The adenosine enclosed by a blue box represents a branch site. **(C)** Schematic representation of the conditional amino acid substitution of the *GluN2A* gene.

**Figure 2:**
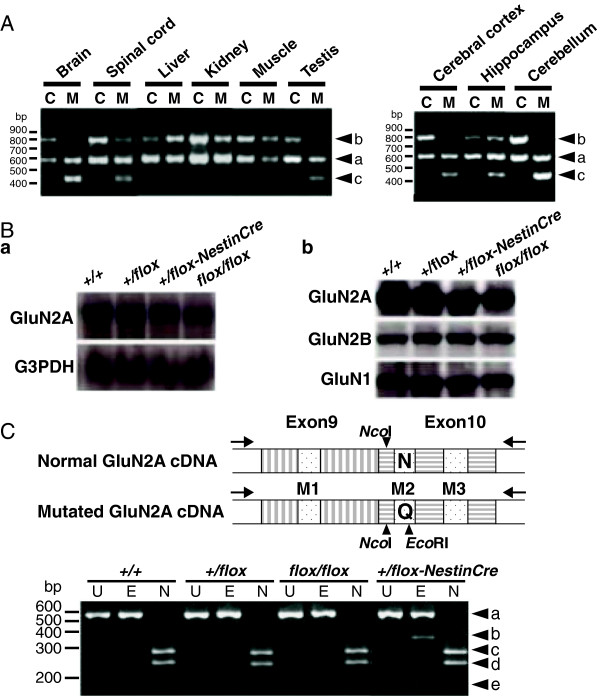
**Cre-loxP recombination and expression of the mutant GluN2A(N595Q) subunit. (A)** PCR-genotyping of various tissues of the adult GluN2A+/flox-non-transgenic (C) and GluN2A+/flox-Cre transgenic (M) mice. The PCR products represent the wild allele (a, 599 bp), flox gene (b, 807 bp), and Cre-mediated-recombinant gene (c, 454 bp). **(B)** The mRNA and protein levels of the GluN2A subunit expressed in the GluN2A+/flox-NestinCre mice are comparable to those of control mice. (B-a) Northern analysis of whole brain RNAs of control (+/+, GluN2A+/+; +/flox, GluN2A+/flox; flox/flox, GluN2Aflox/flox) and GluN2A+/flox-NestinCre mutant mice (+/flox-NestinCre). GluN2A and G3PDH cDNAs were used as probes. (B-b) Western blots of whole brain homogenates of control and mutant mice were performed using rabbit polyclonal antibodies against GluN2A, GluN2B, and GluN1 subunits (Upstate Biotechnology). **(C)** RT-PCR analysis of RNA prepared from whole-brains of control (+/+, wild type; +/flox, GluN2A+/flox-non-transgenic; flox/flox, GluN2Aflox/flox) and GluN2A+/flox-NestinCre mutant (+/flox-NestinCre) mice. Brain RNA was analyzed by RT-PCR amplification followed by digestion of amplified DNAs by NcoI (N), common for both mRNAs, and EcoRI (E), specific for mutant mRNA. Lane U is undigested DNAs. The 521 bp-long PCR product (band a) was the expected size and completely digested by NcoI to predicted lengths (band c: 278 bp, and d: 243 bp), verifying that the PCR products were derived from GluN2A mRNA. Digestion with EcoRI gave rise to two bands (band b: 338 bp, and e: 183 bp) only in the PCR products from the mutant mice (+/flox-NestinCre). There results clearly indicate that detectable mutant GluN2A mRNA was not expressed prior to Cre-loxP recombination and mutant GluN2A mRNA was detectable only in the GluN2A+/flox-NestinCre mice.

We selected GluN2A for the dentate GC-specific abrogation of Mg^2+^ blockade of NMDARs because it is abundantly expressed in the hippocampus of adults, but not during early development [19]. To replace the asparagine residue 595 (N595) with glutamine (N595Q) in the GluN2A subunit, we constructed the targeting vector shown in Figure 1A and Additional file 1: Figure S1. We performed a two-step selection with G418 and Cre recombinase to obtain the desired recombinant ES clones (Figure 1A), and these were used to generate the *flox* homozygous (Figure 1C) mice. The mutually exclusive pre-mRNA splicing of adjacent exons is determined by the proximity of the downstream exon branch point to the upstream exon splice donor site, and a proximity of less than 51 nucleotides completely prevents the adjacent exons from being spliced together [20]. Therefore, the length of the spacer elements between the splice donor site of the wild-type (WT) exon 10 (Figure 1B, *orange box*) and the branching point of the mutant exon 10 (Figure 1B, *green box*) was designed to be 48 nucleotides. If the WT exon 10 between two loxP sequences were deleted by the action of Cre recombinase, the mutant exon 10 would then be spliced into mRNA (Figure 1C). *GluN2Aflox/flox* mice were first crossed with *Nestin-Cre* mice to confirm that the desired recombination event takes place, and their offspring (*GluN2A+/flox-NestinCre*) were subjected to analysis since the majority of homozygous *GluN2A flox/flox-NestinCre* mice died within 2 weeks after birth. In agreement with the distribution of Cre protein (data not shown), Cre-loxP recombination products were observed in the brain and spinal cord (Figure 2A). As expected, there was no significant difference in the size and expression levels of GluN2A mRNA and GluN2A subunits between genotypes (Figure 2B). We next confirmed the exclusive recruitment of WT exon 10 in the *GluN2A flox/flox* mice prior to Cre-loxP recombination and the substitution of WT mRNA (N595) with mutant mRNA (595Q) after Cre-loxP recombination (Figure 2C). These results clearly demonstrate that our method allows the introduction of a desired mutation into any gene of interest in a tissue-restricted manner without altering the level and pattern of target gene expression.

### Induction of the N/Q mutation in the DG

**Figure 3:**
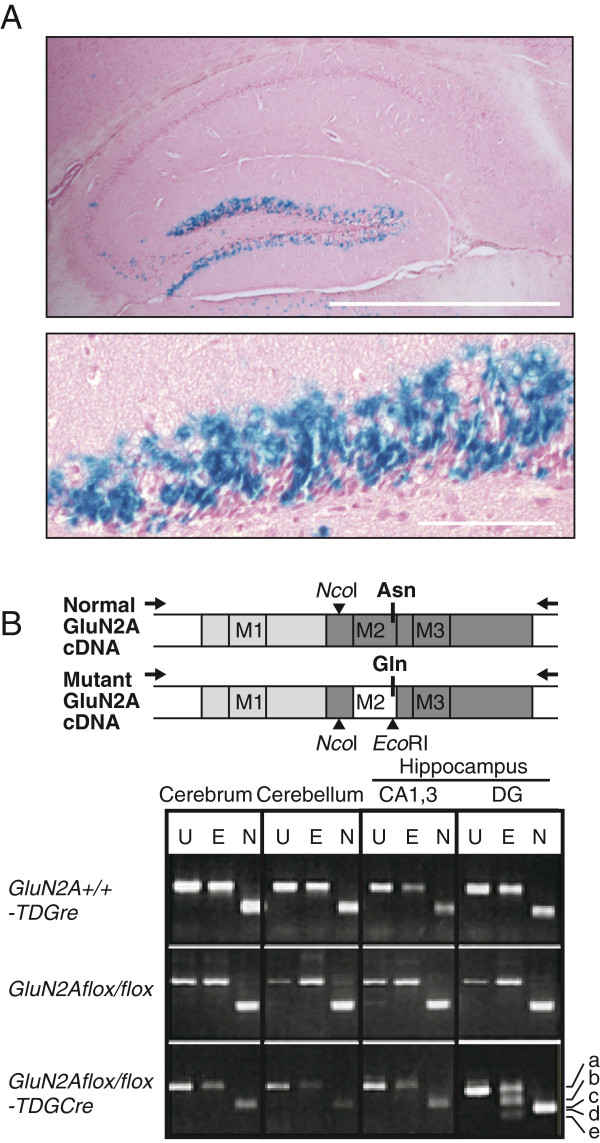
**Tissue-specific expression of the mutant GluN2A(N595Q) subunit. (A)** The lacZ expression in Cre transgenic mice (*TDG-Cre*) was visualized using X-gal staining. Scale bars: 1 mm in the upper panel, 100 μm in the lower panel. **(B)** Schematics of the WT and mutant GluN2A subunits are shown in the upper panel. Transmembrane segments (M1–M3) and the N595Q position (Asn and Gln) in M2 are indicated. RT-PCR analyses of RNA from various brain regions of control (*GluN2A+/+-TDGCre* and *GluN2Aflox/flox*) and mutant mice (*GluN2Aflox/flox-TDGCre*) are shown in the lower panel. The PCR products, 521-bp fragments in WT and mutant cDNAs (band a), were the expected size and completely digested by *Nco*I to the predicted lengths (band c: 278 bp, and d: 243 bp). *Eco*RI digestion resulted in two bands (band b: 338 bp, and e: 183 bp) in the PCR products from the DG of mutant mice, but not from the cerebellum. U, uncut; E, *Eco*RI digestion; N, *Nco*I digestion.

Under control of the Purkinje Cell Protein 2 (PCP2) promoter [21], one of the transgenic (Tag) lines obtained were mice (*TDGCre* mice) expressing Cre recombinase intensively in the dentate GCs of the hippocampus (Figure 3A) and in Purkinje cells of the cerebellum, with slight expression in the thalamus (Additional file 2: Figure S2). These expression patterns became detectable postnatally after approximately 3 weeks and were sustained into adulthood. We crossed *GluN2Aflox/flox* mice with heterozygous *TDGCre* mice to generate *GluN2A+/flox-TDGCre* and *GluN2Aflox/flox-TDGCre* mice. These mice are useful for analyzing the role of GluN2A subunits in the dentate GCs, because (i) the GluN2A subunit is reported to be expressed predominantly in the adult hippocampus and granular layer of the cerebellum, but not in the Purkinje cells, and (ii) NMDAR function in the thalamus is largely dependent on GluN1/GluN2B, GluN2C, and GluN2D complexes [19].

As expected, the Cre-loxP recombination of genomic DNA was selectively induced in the brain, but not in the liver, kidney, muscle, or testis (data not shown). To confirm the switching from wild-type mRNA (N595) to mutant mRNA (595Q) in the DG, brain RNA preparations were analyzed by RT-PCR amplification, followed by digestion of amplified DNA with *Nco*I (common to both mRNAs) and *Eco*RI (specific to mutant mRNA) (Figure 3B). Digestion with *Eco*RI resulted in two bands in the PCR products from the DG of mutant mice, but not from other brain regions (Figure 3B), indicating dentate-specific induction of the N/Q mutation. Consistent with the fact that Cre-recombinase was expressed in nearly half of the dentate GCs in *TDG-Cre* mice (Figure 3A), approximately half of RT-PCR products from the DG of homozygous mice (*GluN2Aflox/flox-TDGCre*) were successfully digested with *Eco*RI (Figure 3B). Notably, we also histologically confirmed that the brains from mutant mice did not show any obvious morphological abnormalities (data not shown).

Based on the above results, we analyzed the physical and neurological features, along with the behavior, of *GluN2A+/flox-TDGCre* and *GluN2Aflox/flox-TDGCre* mice. Homozygous and heterozygous mutant mice were viable, fertile, and appeared grossly normal under standard conditions. No significant effects on physical characteristics, neurological reflexes, and pain sensitivity were observed. Because slight abnormalities in motor function (wire hanging and rotarod tests) were observed in homozygous, but not in heterozygous, mice (Table 1 and Additional file 3: Figure S3), we analyzed the physiological and behavioral functions of heterozygous mice (*GluN2A+/flox-TDGCre* for the mutant and *GluN2A+/flox* for the control) in detail.

**Table 1:** General physical characteristics and sensory-motor functions

	**Controls**	**Mutants**	**p value**
*Physical Characteristics*			
Age (week)	10~13	10~13	
Weight (g) (10~13w)	25.8 (±0.3)	24.7 (±0.3)	0.0153
Weight (g) (14~17w)	30.0 (±0.4)	28.8 (±0.5)	0.244
Whiskers (% with)	79	84	
Fur (% with normal fur)	100	100	
Rectal temperature (°C)	36.5 (±0.2)	36.2 (±0.2)	0.2697

*Sensory Motor Reflex*			
Ear twitch (% with quick response)	89%	95%	
Whisker twitch (% with normal response)	100%	95%	
Righting reflex (% with normal response)	100%	100%	

*Motor tests*			
Wire hang (latency to fall: sec)	55 (±2)	54 (±2)	0.7684

Data represent the mean ± S.E.M. (mutant: *n* = 19, control: *n* = 19).

### Reduction of voltage-dependent Mg^2+^ block of NMDA excitatory postsynaptic currents in the dentate GCs

**Figure 4:**
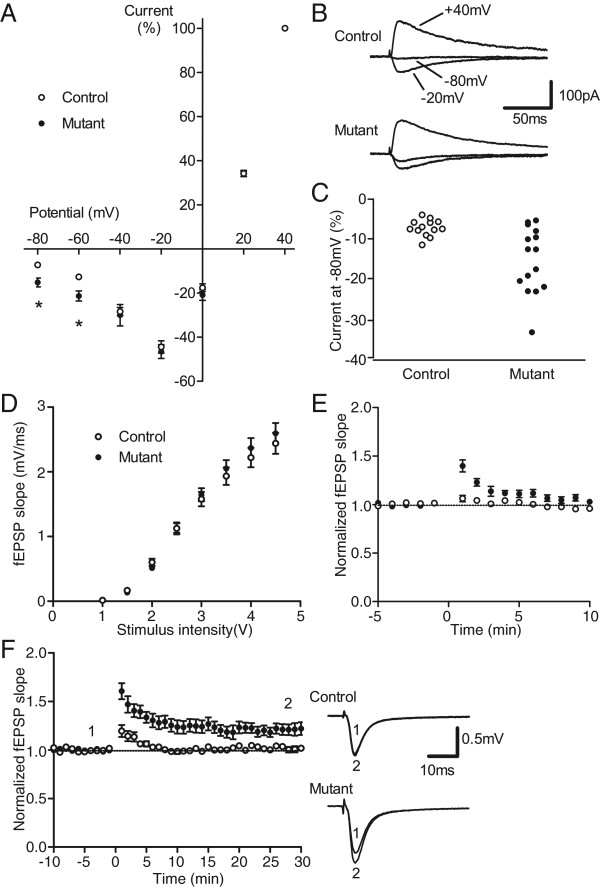
**Altered NMDA EPSCs and synaptic plasticity in the dentate GCs. (A)** Altered current–voltage relationship of NMDA EPSCs in mutant GCs. The amplitudes of NMDA EPSCs at each potential were normalized to that at +40 mV and plotted against the holding potential. Relative EPSC amplitudes at −60 mV and −80 mV were significantly different between control (open circles, *n* = 13) and mutant (filled circles, *n* = 15) mice (*p* < 0.005). **(B)** Sample recordings of NMDA EPSCs at +40, −20, and −80 mV. Stimulus artifacts are truncated. **(C)** Relative amplitudes of NMDA EPSCs at −80 mV. Each symbol represents a single cell. **(D)** Input–output relationship of fEPSPs recorded from the DG (*n* = 15 each). **(E)** Enhanced short-term potentiation in mutant mice. Tetanic stimulation (100 Hz for 0.5 s repeated 4 times) was applied at time 0. The magnitude of potentiation just after tetanic stimulation was significantly larger in the mutants (*p* < 0.001, *n* = 8 each). **(F)** Facilitated induction of LTP in mutant mice. The same pattern of tetanic stimulation as in **(E)** was applied at twice the test intensity. The magnitude of potentiation 25–30 min after the tetanic stimulation was significantly greater in the mutants (*p* < 0.01, *n* = 7 each). Sample recordings on the right were obtained at the times indicated by the numbers. All values are expressed as the mean ± S.E.M. Statistical significance was evaluated using two-tailed Mann–Whitney tests, Student’s *t*-tests, or ANOVA.

To verify the incorporation of the mutant GluN2A subunit into the functional NMDAR in the DG, we recorded excitatory postsynaptic currents (EPSCs) mediated by NMDAR in the dentate GCs. The medial perforant path (MPP) input was activated, and the current–voltage relationship of pharmacologically isolated NMDA EPSCs was examined. While NMDA EPSCs showed the typical J-shaped voltage dependence in the control and mutant mice, the relative EPSC amplitudes at −60 mV and −80 mV were significantly larger in the mutant mice compared to the control mice (Figure 4A and B). Thus, as expected, the voltage-dependent Mg^2+^ block of synaptic NMDARs was reduced in the GCs of the mutant mice. The reduction of the Mg^2+^ block was apparent in approximately half of the recordings (Figure 4C), which correlates with the expression ratio of Cre-recombinase in the GCs shown in Figure 3A.

Next, we recorded field excitatory postsynaptic potentials (fEPSPs) to examine synaptic plasticity at the MPP input in standard physiological saline. The input–output relationship of fEPSPs was indistinguishable between control and mutant mice (Figure 4D). Paired-pulse stimulation induced the depression of fEPSPs, and the depression ratio was indistinguishable between these mice (0.659 ± 0.016, *n* = 15 in controls; 0.659 ± 0.019, *n* = 15 in mutants). Thus, basal synaptic transmission, largely mediated by the AMPA receptor, is apparently not affected in mutant mice. LTP in the DG requires activation of NMDARs and is often difficult to induce in *in vitro* slices with intact synaptic inhibition [22,23]. In our experimental conditions, tetanic stimulation at the test intensity failed to induce lasting potentiation and caused reversible short-term potentiation (STP) in both control and mutant mice. This STP was significantly larger in the mutant mice (Figure 4E). Whereas the tetanic stimulation at the increased stimulus intensity failed to induce LTP in the control mice, it consistently induced LTP in the mutant mice (Figure 4F). These results indicate that the induction of both STP and LTP is facilitated in the DG of the mutants.

### Hippocampal place representation in the mutant mice

**Figure 5:**
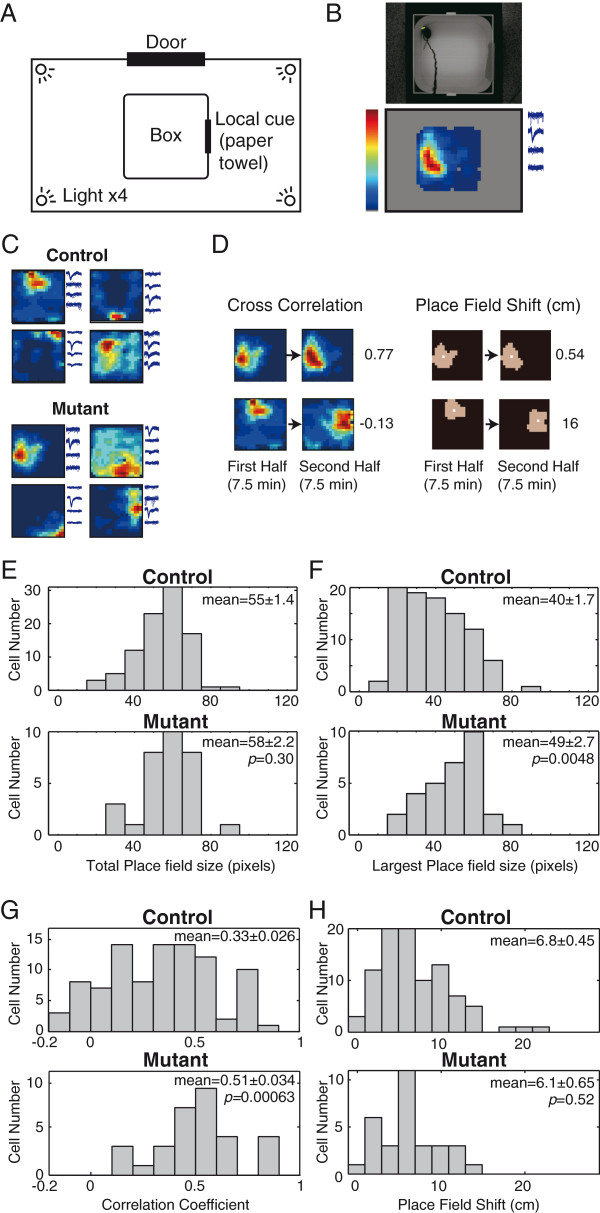
**Recording setup and CA1 place cell firing fields. (A)** Place cells were recorded in a white round-cornered square box (40 × 40 cm, 30 cm high). The box was housed in a 180 × 120 cm soundproof room. A brown paper towel was hung on the wall of the box as a local cue. **(B)** The animal’s position was tracked using a video camera (upper). Color-coded firing rate maps for CA1 cells in the recording box (lower). Red indicates maximum rate, blue indicates silent. Regions not visited by the mouse are shown in gray. Spike waveforms recorded by each electrode are shown in the right side. **(C)** Representative place fields of CA1 place cells in control (upper) and mutant mice (lower). Each recording time was 15 min. **(D)** The correlation coefficient (left) and the value of place field shift (right) between the two place fields is shown at the side of the maps. Brown areas in the right panels indicate place fields (pixels whose firing rates exceed the overall mean firing rate + *SD*). White dots indicate the center of mass of the place fields. Place field shift was defined by the displacement of the center of mass of the place field. **(E)** Histograms of total place field size of CA1 cells in control (upper) and mutant mice (lower). **(F)** Histograms of largest place field size in control (upper) and mutant mice (lower). **(G)** Correlation coefficients of two place fields recorded successively in the same environment are displayed. Each recording time was 7.5 min. **(H)** The shift of place fields recorded successively in the same environment is displayed. Values in panel **(E, F, G, H)** indicate mean ± S.E.M. Significance was evaluated using the Wilcoxon rank sum test.

To test the role of the Mg^2+^ block of DG NMDAR in hippocampal computational function, we analyzed place cell activity in the hippocampus. Place cells are neurons whose firing rate increases when an animal is in a specific location in a given space [14]. Their activity is thought to be involved in spatial navigation. We recorded hippocampal CA1 neurons, the major output of the hippocampal circuit. Mice were allowed to move freely in an open, white, round-cornered square box for a 15-min habituation session once daily (Figure 5A). After at least 7 days of habituation, CA1 place cell activity was recorded in the same box for 15 min (Figure 5B). We recorded the activity of 93 CA1 pyramidal cells and seven interneurons from control mice (*n* = 8) and 31 CA1 pyramidal cells and six interneurons from mutant mice (*n* = 5) under freely moving conditions. We first measured the basic firing properties of pyramidal cells and interneurons during exploratory behavior. As shown in Table 2, the mean firing rate, firing rate variability (measured by the Fano factor), and spike shape of the cell types were not significantly different. These data show that CA1 cells from mutants have similar firing properties to those from control mice.

**Table 2:** Basic firing property of CA1 cells

	**Controls**	**Mutants**	**p value**
*Pyramidal cells*			
Mean firing rate (Hz)	1.22 ± 0.14	1.16 ± 0.21	0.76
Spike width (μs)	691 ± 64.4	685 ± 69.4	0.31
Fano factor (100ms bin)	1.54 ± 0.052	1.44 ± 0.67	0.67

*Interneurons*			
Mean firing rate (Hz)	22.7 ± 9.4	29.8 ± 17.5	0.63
Spike width (μs)	236 ± 45.2	246 ± 41.6	0.69

Data represent the mean ± S.E.M. (mutant: *n* = 93 cells, control: *n* = 31 cells). Significance evaluated by the Wilcoxon rank sum test.

We then assessed the place cell properties of CA1 pyramidal cells in mutant mice. As shown in Figure 5C and Additional file 4: Figure S4, CA1 pyramidal cells in the mutant mice showed robust place-specific discharge. Therefore, the DG-restricted NMDAR mutation did not interfere with place field formation by CA1 cells. To quantitatively compare the specificity of the place fields, we measured the size of the place fields. As shown in Figure 5E, CA1 cells in the mutant mice exhibited no significant change in the size of the firing field. However, we found a difference in the properties of the place fields. In both controls and mutants, many CA1 cells have more than one firing field (Figure 5C and Additional file 4: Figure S4). To explore the properties of the place fields in more detail, we compared the size of the largest firing field (main place field) and the number of firing fields. We found that mutants have a significantly larger main firing field (Figure 5F) and the total number of firing fields was smaller (3.6 ± 0.18 in controls; 2.4 ± 0.20 in mutants, *p* = 0.00052, Wilcoxon). Measurement of the mean running speed revealed no differences between genotypes (5.89 ± 0.91 cm/s for controls, 5.82 ± 0.02 cm/s for mutants, *p* = 0.84, Wilcoxon). Therefore, although running speed can influence place cell activity [24], it fails to explain the observed differences in place cell properties.

### Place fields in the mutant mice were more stable

Because NMDARs have been shown to regulate place field stability [25], one possible mechanism for the larger main place field and the smaller number of firing fields in the mutants is that the temporal fluctuations in the place fields are diminished by the mutation. To test this, we used the Pearson product-moment correlation coefficient for two place fields recorded successively in the same environment (Figure 5D*, left*). Place fields are known to exhibit fluctuations even when the animal is in the same environment [26], and in particular, larger fluctuations are observed in the freely-moving condition than in food-seeking [27]. Place fields of the mutant mice showed higher correlations than control mice (*p* = 0.00063, Wilcoxon) (Figure 5G), indicating that the mutants have more stable place fields. In contrast, the shift in the place fields between the two recordings (Figure 5D*, right*), another measure of similarity between two place fields, exhibited no detectable differences (Figure 5H). These two measures have different properties: place field shift (Figure 5H) is only affected by changes in the high-firing-rate field, whereas the correlations for the place fields (Figure 5G) evaluate the differences between entire place field maps. Therefore, the latter is more sensitive to small changes in place fields. Taking into account the different characteristic of these two measures, the mutation does not affect the persistence of place receptive fields, but reduces small fluctuations in the place fields.

### Impaired separation of place representations in two different environments

**Figure 6:**
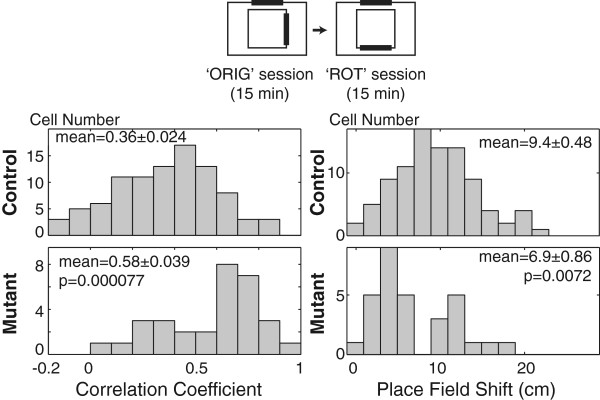
**Pattern separation of CA1 place representation.** Distribution of correlation coefficients (left) and place field shift (right) between the two place fields (“ORIGinal cue” vs. “ROTated cue”) in control and mutant mice. Each recording time was 15 min. Values indicate mean ± S.E.M. Significance was evaluated using the Wilcoxon rank sum test. Values indicate the mean ± S.E.M.

The DG has long been proposed to perform pattern separation, an ability to distinguish between similar input patterns. Therefore, we tested the pattern separation of hippocampal place representation by comparing the place fields of two visually similar but distinct environments. The two testing environments shared the same recording room and box, but the local cue (a paper towel) was rotated 90 degrees clockwise (Figure 6). Place fields were first recorded in the ‘original cue’ condition and then recorded in the ‘rotated cue’ condition. During cue translocation, mice were allowed to rest in their home cage (~1 min). In control mice, most of the cells changed their place fields after local cue rotation (Figure 6, *upper* and Additional file 5: Figure S5*A*, *upper*), which is consistent with previous results in mice [27,28]. In contrast, as shown in cross correlation histograms (Figure 6, *lower-left* and Additional file 5: Figure S5 *A*, *lower*) and place field shift histograms (Figure 6, *lower-right*), more than half of the mutant cells were insensitive to local cue rotation. Statistical analyses confirmed that the place fields of the mutant mice in the two environments were more similar than those of the control mice (cross correlation; *p* = 0.000077, Wilcoxon, and place field shift; *p* = 0.0072, Wilcoxon). We used a distal cue-centric coordinate frame for this analysis, because correlation coefficients for local cue-centric coordinate frames were significantly lower than those for distal cue (recording room, door, etc)-centric coordinates in both controls and mutants (Additional file 5: Figure S5*B* and Additional file 6: Figure S6). These results indicate that place representation in the CA1 of the mutant mice is less sensitive to environmental differences. Another possible interpretation is that attention to local cues was only decreased in the mutants. However, this is unlikely because no significant difference was detected in the correlation coefficient for local cue-centric coordinates between controls and mutants (Additional file 6: Figure S6).

### Learning deficit in the mutant mice

**Figure 7:**
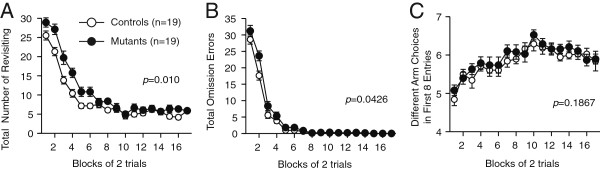
**Performance deficit of the mutants in the eight-arm radial maze test. (A)** Numbers of revisiting errors. **(B)** Total omission errors. **(C)** Numbers of different arms chosen within the first eight choices. Values indicate mean ± S.E.M. Significance was evaluated using two-way repeated-measures ANOVA.

We showed that place representations in the mutants were more stable and less sensitive to environmental differences compared with those of the controls (Figures 5 and 6). To test whether the mutation affects hippocampal-related behavior, we examined the spatial working memory of the mutants. In the eight-arm radial maze test, mutants showed spatial learning deficits in the initial phase of training, but they acquired normal performance in the late phase (Figure 7). This result indicates that the mutants have a slight but significant defect in the performance of a task requiring spatial working memory.

### Adult neurogenesis in the mutant mice

Adult neurogenesis in the DG [29] has been shown to contribute to pattern separation [30-32], and NMDARs are involved in the survival of newborn neurons in the DG [33]. This raised the possibility that the NMDAR mutation might affect the number of adult-born dentate granule cells. To test this, we counted the number of neurons newly produced in the subgranular zone (SGZ) of the DG. There was no detectable difference in numbers of BrdU-positive cells in the SGZ and hilus between the control and mutant mice at 24 h (Additional file 7: Figure S7) and 28 days (data not shown) after BrdU administration. Therefore, impaired pattern separation in our mutant mice was unlikely due to changes in adult neurogenesis.

## Discussion

Studies of gene function using gene-manipulation techniques have allowed many breakthroughs in the biological sciences, including neuroscience. Here we introduced a mutation into the *GluN2A* gene by applying regulatory splicing mechanisms, in conjunction with Cre-loxP recombination, to elucidate the function of NMDARs in the DG of the hippocampus. Our system is widely applicable and has great advantages for introducing desired mutations into genes of interest in a spatiotemporally restricted manner, without altering the level and pattern of the expression of the relevant gene. Thus, this method is expected to open up new possibilities for studying the physiological functions of critical amino acid residue(s) in target genes *in vivo*.

NMDARs are predicted to be tetramers composed of two GluN1 and two GluN2 subunits [34]. We estimated the composition of NMDARs in the heterozygous mutant mice according to Seeburg’s prediction [35]. Twenty-five percent should be WT receptors (2 × GluN1-2 × GluN2A^N^) with normal Ca^2+^ conductance and voltage-dependent Mg^2+^ block. Fifty percent should be hybrid receptors comprised of one WT and one mutated subunit (2 × GluN1-GluN2A^N^-GluN2A^Q^), and the remaining 25% would be purely mutant receptors containing two mutated subunits (2 × GluN1-2 × GluN2A^Q^) (Additional file 8: Figure S8). Therefore, 75% of GluN1-GluN2A receptors in a single Cre-expressing cell may contain mutant GluN2A subunits, and such mutations should be expressed in nearly half of the GCs in the DG as described above (Figure 3A). Because the Mg^2+^ block of NMDA EPSCs was altered as predicted, our genetic manipulation indeed modified synaptic NMDARs in the GCs (Figure 4). Previous studies have analysed the effects of modulating the Mg^2+^ block. Overexpression of Mg^2+^ block-deficient NMDARs has been shown to impair long-term memory in *Drosophila*[7]. In addition, elevating extracellular Mg^2+^ levels leads to an increase in LTP in cultured hippocampal neurons [6,36] or in the CA1 region of hippocampal slices from rats receiving magnesium supplements [6,36] and improves learning and memory [6,36]. The present study demonstrates that reduced Mg^2+^ block facilitates STP and LTP at the PP-DG synapse in mouse hippocampal slices, which is apparently contradictory to previous observations in rats. Intriguingly, the mutation increased NMDAR currents only at −60 to −80 mV range (Figure 4C). This suggests that facilitated synaptic potentiation is likely due to this enhancement of NMDAR currents near the resting potential, but not at the potential range for coincidence detection (> −30 mV). One possible mechanism for this is that the enhanced NMDAR currents in the basal condition promote the tetanus-induced activation of intracellular signaling pathways required for synaptic potentiation, possibly via enhanced Ca^2+^ influx. Another possibility is that the increased NMDAR current enhances depolarization during tetanic stimulation, thereby leading to the facilitation of synaptic potentiation.

To explore the role of the Mg^2+^ block of NMDARs in hippocampal information processing, we analyzed the place cell activity of hippocampal CA1 cells. In spite of the significant change in electrophysiological properties in the dentate GCs (Figure 4), mutant mice exhibited normal place-specific discharges (Figure 5C and Additional file 4: Figure S4). The CA1 receives synaptic input directly from the entorhinal cortex (EC), as well as indirectly from the EC through the DG, and previous lesion experiments have shown that the direct EC-CA1 pathway is sufficient for forming the CA1 place field [37]. In the mutant mice, mutant GluN2A was not expressed in the EC (Additional file 2: Figure S2), thus the direct EC-CA1 pathway remains intact. This may explain why the DG-restricted mutation does not affect place field formation in the CA1. Despite normal place field activity in the mutants, the number of recorded cells was smaller than that of controls (11.6 cells/animal for controls, and 6.2 cells/animal for mutants). This raised the possibility that the ratio of active cells to entire cells in a given environment was lower in the mutants. Because a much larger portion of hippocampal neurons are active during sleep and anesthesia than in the awake exploring animal [38], comparing activity during sleep and exploring would provide information on the population of cells active during exploration. However, we unfortunately did not record activity during rest/sleep in the present study, so we are unable to estimate the proportion of behaviorally active cells. Therefore, we cannot rule out the possibility that the mutation decreased the population of cells active during exploration.

Although the place fields of the mutants appeared normal, we found some differences in the dynamic properties of the place fields. Specifically, the CA1 place fields in the mutants showed reduced fluctuations (Figure 5G), and the place fields for the two different environments are more similar in the mutants than in the controls (Figure 6). The DG has been considered a site of pattern separation [39], and the latter result presented here is consistent with this hypothesis. McHugh et al. (2007) have also shown impaired pattern separation in animals lacking functional NMDARs in the DG [40]. It is interesting to note that both the hyperfunction of NMDARs due to the loss of the Mg^2+^ block (Figure 4) and the loss of NMDARs in the DG [40] can cause impairments in pattern separation. This raises the possibility that there exist a range of NMDAR activity optimal for the function of pattern separation.

The expression pattern of Cre in the DG (Figure 3A) and the results of the *in vitro* electrophysiological experiment (Figure 4C) demonstrate that the GCs of the mutants were mosaic for mutated GluN2A expression. However, the activity of each CA1 cell is governed by a large number of GCs: each CA3 cell receives contacts from approximately 50 GCs, and a single CA1 neuron receives inputs from more than 5000 CA3 pyramidal cells [41]. Therefore, the effect of this mosaicism in the mutants would be averaged out on CA1 cells.

Although NMDARs have classically been considered not to be functional in cerebellar Purkinje cells [42,43], a recent report suggests that the NMDARs in the cerebellar Purkinje cells of adult rodents contribute to plasticity at the synapses between climbing fibers and Purkinje cells [44]. In our mutant mice, Cre/loxP recombination was observed in the cerebellum (Figure 2A), and slight motor abnormalities were found in homozygous mice (see Results), suggesting the possibility that mutated GluN2A in the cerebellum may contribute to the mutant phenotype even in heterozygotes. The cerebellum is known to process self-motion signals [45,46], and a recent report showed that a transgenic mouse line with impaired protein kinase C-dependent plasticity at parallel fiber–Purkinje cell synapses has impaired hippocampal place field activity under specific circumstances, i.e., when animals rely on self-motion cues, or when there is conflict between visual cues and self-motion cues [47]. This report indicated a role for the cerebellum in processing self-motion signals for shaping hippocampal place field activity. In our place cell experiment, visual cues were always presented, and there was no conflict between visual cues and self-motion cues in the recording sessions (Figures 5 and 6). Therefore, it seems unlikely that mutated GluN2A in the cerebellar Purkinje cells is involved in the place cell abnormalities described here, but we cannot rule out the possibility that NMDARs in cerebellar Purkinje cells contribute to place cell activity during visually guided locomotion.

How does the mutation cause these changes in place cell properties? When the animal is exposed to a different environment, spatial maps change completely (“remapping”) [48]. Each spatial map is thought to be an attractor state in the recurrent network, and possibly resides in the CA3 [49,50]. Recurrent networks can have a finite number of stable firing patterns, and they tend to move to and settle into these stable firing patterns. In support of this idea, the activity of the CA1 depends upon the output of the CA3, and has been shown to change abruptly when the input changes gradually [16]. In such a discrete output system, a small input difference is usually ignored in nature. However, the addition of an appropriate amount of noise to the input of the system can assist in state transitions that result from subthreshold differences in input. This noise-assisted enhancement of input sensitivity is called stochastic resonance and has been found in several sensory systems [51,52]. Interestingly, hippocampal place fields exhibit large fluctuations even when the animal remains in the same environment [26], and these fluctuations are not due to random variation, but can be interpreted as switching between multiple spatial maps [53]. This raises the possibility that place field fluctuations serve to output different patterns in response to small input differences. Therefore, if the mutants have difficulty switching between spatial maps, their place fields would be stable and pattern separation would be impaired. If so, the next question is how a reduction in the Mg^2+^ block in the mutants impairs spatial map switching. A previous theoretical study has suggested that large NMDA EPSCs and the facilitated STP and LTP observed in our mutant mice can inhibit attractor state switching [54]. The CA3 has been regarded as the primary attractor network in the hippocampus because of its extensive recurrent collaterals [50]. Feedback connections from hilar mossy cells to the dentate GCs are another candidate for the attractor network in the hippocampus. Therefore, NMDARs in the dentate GCs could modulate attractor network properties [55]. Using neural network simulations, Rolls et al. 2008 reported that an increase in NMDAR conductance stabilizes attractor states and makes it more difficult to switch to another state in response to a new external stimulus. Thus, the reduced Mg^2+^ block in the GCs of our mutant mice may stabilize the spatial map by increasing NMDAR conductance.

The DG is one of the two brain regions where adult neurogenesis occurs [29,56]. Recently, young adult-born GCs in the DG were shown to specifically mediate pattern separation [32]. In our mutant mice, the mutated version of GluN2A was expressed in mature GCs, which constitute the majority (~95%) of total GCs (Figure 3A), but we did not investigate whether mutated GluN2A is also expressed in young GCs. Determining the cell type responsible for abnormalities in place cell properties in the mutants is a goal for future experiments.

We showed that the reduced Mg^2+^ block of the NMDARs in the DG stabilizes place fields and impairs pattern separation (Figures 5 and 6). The former would be advantageous to learning, but the latter would not. As stated above, if spatial map switching is the basis for both place field stability and pattern separation, better pattern separation would be inherently incompatible with high place field stability. Considering this trade-off, the spatial working memory deficit in the mutants (Figure 7) suggests that the Mg^2+^ block in the dentate gyrus regulates hippocampal spatial information processing to maximize learning ability.

## Conclusions

Here we found that the N595Q substitution in GluN2A reduced the Mg^2+^ block of NMDAR-mediated synaptic currents in dentate granule cells and facilitated activity-dependent synaptic potentiation at the medial perforant path–granule cell synapses. Hippocampal place representation in the mutants was more stable than that in the controls and less sensitive to visual differences. These results imply that the enhanced synaptic potentiation resulting from the reduction of the Mg^2+^ block stabilizes place representation but impairs pattern separation. The mutants also showed working memory deficits, indicating that the Mg^2+^ block contributes to spatial learning.

## Methods

### Targeting construct design

Because N595 is located in exon 10 of the *GluN2A* gene, [57], we generated a mouse line (*GluN2Aflox*) harboring a tandem array of the wild-type (WT) exon 10 encoding N595 and a mutant exon 10 encoding Q595 separated by a 70-nucleotide (nt) artificial intron. This artificial intron was comprised of a 5′-donor sequence (9 nt) and a 3′-acceptor sequence, including the branch point (27 nt) from the mouse β-globin intron and loxP sequences (34 nt) that were inserted between the donor and acceptor sequences (Figure 1B). The length of the spacer elements between the splice donor site of the WT exon 10 (Figure 1B*, orange box*) and the branch point of the mutant exon 10 (Figure 1B*, green box*) was designed to be 48 nt. However, it is possible that on occasions, the 5′ normal exon will be skipped due to unforeseen mechanisms. In order to ensure that the normal exon 10 is exclusively spliced into the mRNA, the splicing acceptor element of normal exon 10 was substituted with a 73-nt Sxl-binding splicing acceptor sequence that is preferentially selected among mutually spliced exons in the absence of Sxl protein (notably, Sxl-like protein has not been found in mice) [58] (Figure 1B*, violet*). The Neo-TK cassette, consisting of neomycin-resistant and thymidine-kinase genes flanked by loxP sequences, was located on the 5′ side of the Sxl-binding splicing acceptor sequences (Figure 1A and B). Two-step selection was performed to obtain the desired recombinant ES clones. The targeting vector was electroporated into TT2 ES cells [59]. After G418 selection, one homologous recombinant clone (out of 399 G418-resistant clones) was identified. This clone was expanded, transfected with a Cre recombinase-expression plasmid (pIC-Cre), and further selected with gancyclovir. We confirmed that one clone out of 216 gancyclovir-resistant clones exhibited precise excision of the loxP-Neo-TK-loxP cassette by Cre-loxP recombination. This secondary recombinant ES clone was then microinjected into 8-cell embryos to generate flox homozygous (*GluN2Aflox/flox*) mice using standard procedures, and was confirmed by Southern blot analyses.

### PCR analysis for genotyping and Cre-loxP recombination

After the fidelity of PCR genotyping for GluN2A was confirmed, subsequent genotyping performed by PCR using primers Pr137: 5′GTGGTAAAATCCAGTTAGATAG3′ and E1R: 5′GGGTTATAGAATGGATGGTTA3′. PCR conditions were as follows; denaturation at 94°C for 1 min, followed by 30 cycles of 1 min at 94°C, 1 min at 60°C, 1 min at 72°C, a final extension at 72°C for 5 min, and storage at 4°C. The floxed, wild-type and Cre-mediated recombined GluN2A genes correspond to PCR products of 807, 599 and 454 bp, respectively. For analysis of Cre-loxP recombination in various tissues by PCR, genomic DNA samples from various tissue were subjected to the same PCR condition as above, using primers CRE10: 5′CAACGAGTGATGAGGTTCGCAA3′ and CRE13: 5′CCCCAGAAATGCCAGATTACGT3′.

### RT-PCR analysis

The GluN2A cDNA fragments were amplified using total RNA by RT-PCR with primers mE1F: 5′ATCAACGAGCAGTTATGGCC3′ and mE1R: 5′GTCATGAGGTCTCTGGAACT3′. The amplified DNA fragment of 521 bp was digested by EcoRI or NcoI.

### Generation of transgenic mice

Nestin-Cre and TDG-Cre transgenic lines were generated. The Nestin-Cre mice were produced carrying tandem arrays of the Cre-recombinase gene driven by the rat nestin promoter, the internal ribosome entry sites (IRES) sequence, lacZ gene, and a neuron specific enhancer [60]. In TDG-Cre we used a Purkinje Cell Protein 2 (PCP2) promoter to drive the expression of Cre-recombinase. We used a transgenic strain, CAG-CAT-Z, as a reporter line [61].

### Neurological screen

A neurological screen was conducted as previously described [62]. The righting, whisker touch, and ear twitch reflexes were evaluated. A number of physical features were also recorded, including the presence of whiskers or bald patches in the hair.

### Wire hanging test

Neuromuscular strength was tested with the wire hanging test. The mouse was placed on a wire mesh and the mesh was then inverted, to force the animals to grip the wire. Latency to fall was recorded, with a 60 sec cut-off time.

### Rotarod test

Motor coordination and balance were tested using an accelerating rotarod (UGO Basile Accelerating Rotarod). The test was performed by placing a mouse on a rotating drum (3 cm diameter) and the time each animal was able to maintain its balance on the rod was measured. The speed of the rotarod accelerated from 4 to 40 rpm over a 5-min period.

### Hot plate test

The hot plate test was used to evaluate sensitivity to a painful stimulus. Mice were placed on a 55.0 (±0.3) °C hot plate (Columbus Instruments, Columbus, Ohio), and latency to the first hind-paw response was recorded. The hind-paw response was either a foot shake or a paw lick.

### Slice electrophysiology

Adult male mutant and control mice (14–17 weeks old) were used. Mice were decapitated under halothane anesthesia and both hippocampi were isolated. Transverse hippocampal slices (370–400 μm) were obtained using a tissue slicer (VT1000S; Leica Microsystems, Nussloch, Germany or Vibratome 3000 plus; Vibratome Company, St. Louis, MO, USA) in sucrose-containing saline composed of (in mM): sucrose, 72; NaCl, 80; KCl, 2.5; NaH_2_PO_4_, 1.0; NaHCO_3_, 26.2; glucose, 20; CaCl_2_, 0.5; and MgCl_2_, 7 (equilibrated with 95% O_2_/5% CO_2_) for whole-cell recordings or in standard saline (see below) for field potential recordings. Slices were incubated for 30 min at 30°C in a standard saline solution composed of (in mM): NaCl, 125; KCl, 2.5; NaH_2_PO_4_, 1.0; NaHCO_3_, 26.2; glucose, 11; CaCl_2_, 2.5; and MgCl_2_, 1.3 (equilibrated with 95% O_2_/5% CO_2_) and maintained in a humidified interface holding chamber at room temperature (24–27°C) before use. Electrophysiological recordings were made from slices placed in a submersion-type chamber superfused at 2 mL/min. The bath temperature was maintained at 26.5–27.5°C using an automated temperature controller.

Whole-cell recordings were made from GCs in the DG using a blind whole-cell patch-clamp technique. Voltage-clamp recordings were made with a pipette filled with a solution composed of (in mM) CsCl, 140; HEPES, 20; NaCl, 8; MgATP, 2; Na_2_GTP, 0.3; EGTA, 0.5; and CaCl_2_, 0.05 (pH adjusted to 7.3 with CsOH). The recording pipette was placed in the middle third of the GC layer. Synaptic currents were evoked at 0.2 Hz by bipolar tungsten stimulating electrodes that were placed in the middle third of the dentate molecular layer to activate the input from the medial perforant path (MPP). Activation of MPP input was verified by the depression of synaptic currents in response to paired stimuli. EPSCs mediated by NMDARs were recorded in standard saline solution supplemented with 6-cyano-7-nitroquinoxaline-2,3-dione (CNQX, 20 μM), picrotoxin (100 μM), and SR95531 (2 μM). EPSC amplitudes were measured on analysis. Series resistance was monitored during the recordings, and data deviating by more than 15% were excluded. fEPSPs at the MPP synapse were recorded in the middle third of the molecular layer using a glass pipette filled with 2 M NaCl. The MPP was stimulated at 0.05 Hz in standard saline. The initial slopes of fEPSPs were measured.

All recordings were done using a Multiclamp 700B amplifier (Axon Instruments, Union City, CA, USA), filtered at 2–5 kHz, and stored in a personal computer via an interface (digitized at 10 kHz). CNQX and d-2-amino-5-phosphonovaleric acid (d-APV) were purchased from Tocris (Bristol, UK). Picrotoxin was from Wako Pure Chemical Industries, Ltd. (Osaka, Japan). SR95531 was from Sigma (St. Louis, MO, USA). All values are expressed as the mean ± S.E.M. Statistical significance was evaluated using two-tailed Mann–Whitney tests, Student’s *t*-tests, or ANOVA.

### *In vivo* electrophysiology

All procedures were approved by our Institutional Animal Care and Use Committee. Adult male mutant and control mice (14–21 weeks old) were used. The control mice consisted of six WT mice (*GluN2A+/+*) and two floxed mice (*GluN2A+/flox*). We did not detect any significant differences in firing properties and place field measurements between the WT and floxed animals.

Animals were chronically implanted with a miniature microdrive equipped with four electrodes (Neuralynx, Tucson, AZ). Each electrode was composed of four individually insulated tungsten wires (12.7 μm diameter) that were twisted together. The impedance of the electrodes was approximately 400 kΩ at 1 kHz. Animals were anesthetized with a mixture of 0.9% ketamine and 0.2% xylazine. The skull was exposed, and four stainless screws were inserted into the bone for structural support. A small hole (<0.5 mm) was made over the left hemisphere (1.5 mm lateral to the midline, 2.0 mm posterior to the bregma), and the electrodes were inserted through the hole. The electrode tips were positioned 0.9 mm deep from the brain surface. The space between the bone and the microdrive was filled with petroleum jelly, and the microdrive and the bone were glued using dental acrylic.

The electrodes were connected to a unity gain buffer consisting of an operational amplifier (TL074; Texas Instruments, Dallas, TX). Signals from the buffer amplifier were amplified (×3000), and filtered between 300 Hz and 5000 Hz. Amplified signals were sampled by a data acquisition card (PCI-6259; National Instruments, Austin, TX) at 20 kHz. The location of the animal was tracked using an overhead video camera, which tracks a pair of infrared LEDs placed on the top of the microdrive. Neural waveforms and video images were processed and stored using custom software written in LABVIEW7 (National Instruments, Austin, TX).

At least one week before surgery, animals were handled daily and allowed to explore the testing platform (a white, round-cornered square box, 40 × 40 cm, 30 cm high) for 15 min each day. The box was housed in a 180 × 120 cm soundproof room. A brown paper towel was hung on the wall of the box as a local cue. Four incandescent bulbs in 15-cm hemispherical reflectors were mounted in the corners of the room (Figure 5A). One week after surgery, electrodes were screened daily for complex spike cells. Complex spike cells were identified as those that had both a wide peak-to-trough width (>0.3 ms) and complex spike bursts. If no complex spike cell was found, electrodes were lowered by 40 μm and were screened the next day until complex spike cells were found. The recording session began only if complex spike cells persisted for at least 1 day. The animals were exposed to both cue configurations (‘orig’ and ‘rot’) for 15 min each during the cell screening period (11–19 days, depending on individuals) and recording sessions (5 days). The complex spiking cells recorded in the CA1 appear to be pyramidal cells [63,64]; thus, we designated the complex spiking cells in the CA1 as pyramidal cells. The data were excluded if the mouse crossed less than 90% of the platform in a single session or the mean firing rates were below 0.1 Hz. At the end of the recordings, marker lesions were made at the electrode tips by passing a current (20 μA, 10 s). The location of a recording site was determined by estimating the distance along the electrode track associated with the microelectrode position at the time of recording.

### Spike data analyses

Recorded neural waveforms were digitally high-pass filtered above 800 Hz. Spikes with amplitudes 3.5 times greater than noise were extracted for further analyses. The spikes were segregated into multiple single units using automatic clustering software Klustakwik [65] and manual cluster cutting software Klusters [66]. The presence of a refractory period in isolated units was inspected visually by using Klusters. Units with a clear refractory period (>2 ms) in their autocorrelograms were included in the present analysis. The firing rate maps of the complex spike cells were constructed by finding the spike number at each location bin (2 cm × 2 cm) divided by the dwell time in the bin. To eliminate nonspecific firing at rest, spikes occurring when the animal's velocity was less than 1 cm/s were filtered out [28]. The maps were smoothed using a Gaussian spatial and temporal filter with a standard deviation of 1 pixel (2 cm) and 2.5 s, respectively. A place field was defined as a group of at least one contiguous pixel in which the firing rates exceeded the overall mean firing rate plus one standard deviation. “Main place field” was defined as the largest place field for each cell. Signal processing and statistical analyses were performed using MATLAB 6.5 (Mathworks, Natick, MA) and GraphPad Prism5 (GraphPad Software, La Jolla, CA).

### Eight-arm radial maze test

All procedures were approved by our Institutional Animal Care and Use Committee. Adult male mutant and control mice (10–13 or 17–21 weeks old) were used. Mice were housed in a room with a 12-h light/dark cycle (lights on at 7:00 a.m.) with free access to food and water. Behavioral testing was performed between 9:00 a.m. and 6:00 p.m. After the tests, all apparatus were cleaned with super hypochlorous water to remove the scent of mice. The eight-arm radial maze test was conducted in a manner similar to that described previously [62]. The floor of the maze consisted of white plexiglass, and the wall (25 cm high) consisted of transparent plexiglass. Each arm (9 × 40 cm) radiated from an octagonal central starting platform (perimeter 12 × 8 cm) like the spokes of a wheel. Identical food wells (1.4 cm deep and 1.4 cm in diameter) with pellet sensors were placed at the distal end of each arm. The pellet sensors were able to automatically record pellet intake by the mice. The maze was elevated 75 cm above the floor and placed in a dimly lit room with several extra-maze cues. During the experiment, the maze was maintained in a constant orientation. One week before pre-training, animals were deprived of food until their body weight was reduced to 80–85% of the initial level. Pre-training started on day 8. Each mouse was placed in the central starting platform and allowed to explore and consume food pellets scattered on the whole maze for a 5-min period (one session per mouse). After completion of the initial pre-training, mice received another pre-training to take a pellet from each food well after being placed at the distal end of each arm. A trial was finished after the subject consumed the pellet. This series of experiments was repeated eight times, using eight different arms, for each mouse. After these pre-training trials, the actual maze acquisition trials were performed. All eight arms were baited with food pellets. Mice were placed on the central platform and allowed to find all eight pellets within 25 min. A trial was terminated immediately after all eight pellets were consumed or 25 min had elapsed. An “arm visit” was defined as traveling more than 5 cm from the central platform. The mice were confined in the center platform for 5 s after each arm choice. The animals went through one trial per day (34 trials in total). For each trial, choices of arms, latency to get all pellets, distance traveled, number of different arms chosen within the first eight choices, the number of revisiting, and omission errors were automatically recorded. Data acquisition, control of guillotine doors, and data analyses were performed using Image RM software written by Tsuyoshi Miyakawa (available through O’Hara & Co., Tokyo, Japan). The software is based on NIH Image (http://rsb.info.nih.gov/nih-image/). Statistical analyses were conducted using StatView (SAS Institute).

### BrdU labeling and immunohistochemistry

To assess the effects of GluN2A mutations on the number of BrdU-positive cells, mice (18 weeks old) were administered BrdU, 4 × 75 mg/kg i.p., dissolved in saline, every 2 h. Mice were sacrificed 24 h after the last BrdU injection. After anesthesia, mice were transcardially perfused with 4% paraformaldehyde and brains were collected for immunohistochemistry. All brains were post-fixed overnight in 4% paraformaldehyde at 4°C and soaked in a series of 10%, 20% and 30% sucrose at 4°C. Serial sections of the brains (30 μm sections) were cut through the entire hippocampus on a cryostat. DNA denaturation was conducted by incubation for 2 h in 50% formamide/2× SSC at 65°C, followed by several rinses. Sections were then incubated for 30 min in 2 N HCl and then for 10 min in boric acid. After washing with PBS, sections were incubated in 3% H_2_O_2_ for 30 min to eliminate endogenous peroxidases. After blocking with 3% normal goat serum in 0.01% Triton X-100, cells were incubated with anti-mouse BrdU (1:100; Becton Dickinson) overnight at 4°C. Sections were then incubated for 1 h with secondary antibody (1:1000; biotinylated goat anti-mouse; Vector Laboratories, Burlingame, CA) followed by amplification using an avidin-biotin complex (Vector Laboratories) and visualization with DAB (Vector Laboratories).

## Abbreviations

NMDAR: N-methyl D-aspartate receptor; DG: Dentate gyrus; GC: Granule cells; WT: Wild type; STP: Short-term potentiation; LTP: Long-term potentiation; SGZ: Subgranular zone; BrdU: 5-bromo-2′-deoxyuridine.

## Competing interests

The authors declare no competing financial interests.

## Authors’ contributions

YN and Y-IN developed the gene-manipulation method. YN, EE, SN, YM, TS, TN, JM, MM and Y-IN generated the knock-in mice. YH and KF performed *in vivo* electrophysiology experiments. KK and HS performed slice electrophysiology experiments. TM, KoT and KeT performed behavioral experiments. YH, YN, KK, TM and Y-IN designed and wrote the paper. All authors read and approved the final manuscript.

## Supplementary Material

Additional file 1: Figure S1 Genaration of pGETV targeting construct. The loxP sequences (red), the cassette containing the neomycin-resistant and thymidine kinase genes (Neo-TK), the diphtheria toxin A frangment (DT-A) the 5′- and 3′- homologous regions (A, B, and C in bold white letters on black background) of the mouse GluN2A gene, exon9 (light blue), WT exon10 encoding asparagine595 (orange), mutant exon10 encoding glutamine595 (green), the fruit fly *tra* intron1 (violet), and the mouse β-globin intron (blue) are shown. To clearly illustrate each step of vector construction, the figure shows the relative locations of fragments and relevant restriction sites. The length of each fragment is indicated beneath each fragment. Click here for file

Additional file 2: Figure S2 Expression of Cre recombinase in the TDG-Cre mouse. The lacZ expression was visualized using X-gal staining. (A) Whole-brain, (B) cerebellum, (C) thalamus. DG: dentate gyrus, Th: thalamus. Scale bars: 100 μm. Click here for file

Additional file 3: Figure S3 Rotarod and hot plate tests performance. (A) In the rotarod task, the performance of the mutants was slightly worse than control mice, though this failed to raech significance (day1 (1~3trial): p=0.3247, day2 (4~6trial): p=0.0665). (B) In the hot plate test, there was no difference in the response latency between genotypes. Click here for file

Additional file 4: Figure S4 Place field maps of pyramidal cells in CA1 region. Place field maps of all CA1 pyramidal cells recorded from control and mutant mice. Maps are sorted by the place field size. Spike waveforms recorded by tetrode were shown on the right side of each place field map. Maximum firing rates were shown at the upper-right of the map (Hz). Click here for file

Additional file 5: Figure S5 Modulation of place fields in response to cue rotation. (A) Place field maps of all CA1 pyramidal cells recorded in both original cue environment “ORIG” (left) and rotated cue environment “ROT” (right). Recording time was 15 min each. Maps are sorted by the correlation coefficient between the maps recorded in the two environments. (B) Correlation coefficients between the maps for “ORIG” and “ROT” associated with a 0°, 90°, 180°, or 270° CW rotation of the maps for “ROT”. (C) Correlation coefficients are displayed for two place fields recorded successively in the same environment (1st half of ORIG-2nd half of ORIG) and in cuerotated environment (2nd half of ORIG-1st half of ROT). To quantatevely compare these two conditions, correlation coefficients were calculated for 7.5 min of recordings. Bars indicate mean value and error bars indicate *SE*. Two-way ANOVA was used for comparisons between groups, followed by a Bonferroni posttest. Asterisks indicate the relative level of the p-value. * *p* < 0.05, ** *p* < 0.01. Click here for file

Additional file 6: Figure S6 Place field similarity measurements in distal cue- and local cue-centric coordinate frames. Correlation coefficiens between the two place fields (“original cue” vs “rotated cue”) in control and mutant mice were calculated using distal cue (recording room, door, etc)- and local cue (paper towel)-centric coordinate frames. Data are shown in mean ± SE. Significance was evaluated using the Wilcoxon rank sum test. *p = 0.0016; ** *p* = 9.0 ×10^-7^. Click here for file

Additional file 7: Figure S7 The number of BrdU-positive cells in the DG. BrdU-positive cells in the subgranular zone of the DG and hilus are not detectably different in the mutant mice and control mice 24 h after BrdU administration. Click here for file

Additional file 8: Figure S8 NMDA receptors formed with the wild type GluN2A (N) or the mutant GluN2A(Q) subunit. In Cre-expressing cells of *GluN2A+/flox* mice, NMDA receptors will consist of the three populations (middle column). Left column and right column shows the *GluN2A+/flox* and *GluN2Aflox/flox-TDGCre* mice respectively. Click here for file
