# Supplementary figures and images for: Enhanced stability of hippocampal place representation caused by reduced magnesium block of NMDA receptors in the dentate gyrus
Source: Mol Brain. 2014 Jun 4;7:44. doi: 10.1186/1756-6606-7-44 (PMC4073519; doi:10.1186/1756-6606-7-44)

Figure S1 A-D

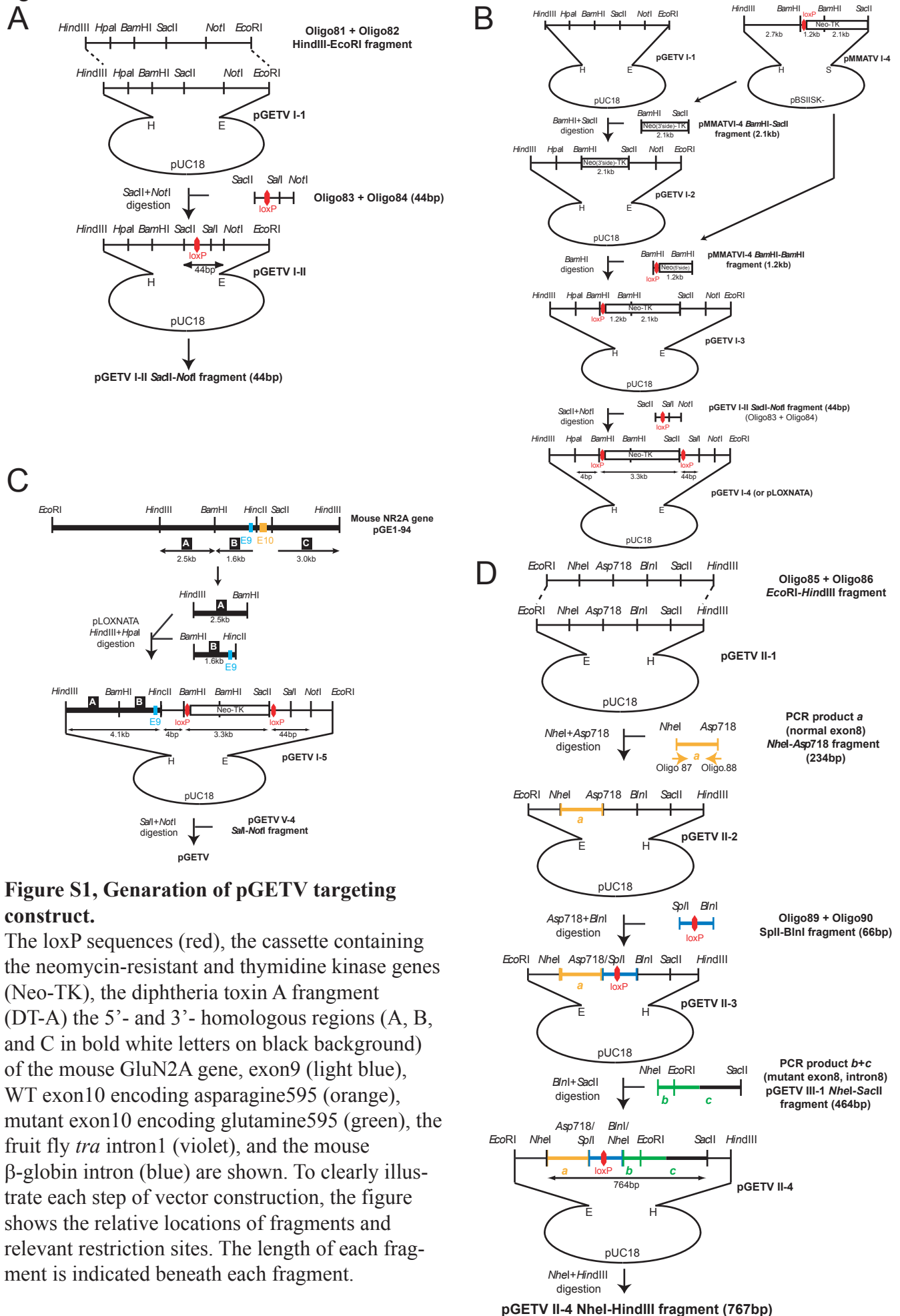

Supplement: Additional file 1: Figure S1 — Genaration of pGETV targeting construct. The loxP sequences (red), the cassette containing the neomycin-resistant and thymidine kinase genes (Neo-TK), the diphtheria toxin A frangment (DT-A) the 5′- and 3′- homologous regions (A, B, and C in bold white letters on black background) of the mouse GluN2A gene, exon9 (light blue), WT exon10 encoding asparagine595 (orange), mutant exon10 encoding glutamine595 (green), the fruit fly tra intron1 (violet), and the mouse β-globin intron (blue) are shown. To clearly illustrate each step of vector construction, the figure shows the relative locations of fragments and relevant restriction sites. The length of each fragment is indicated beneath each fragment. [file 1756-6606-7-44-S1.zip › 1850664515130282_add1/1850664515130282_add1.pdf]

Figure S1 E-H

E

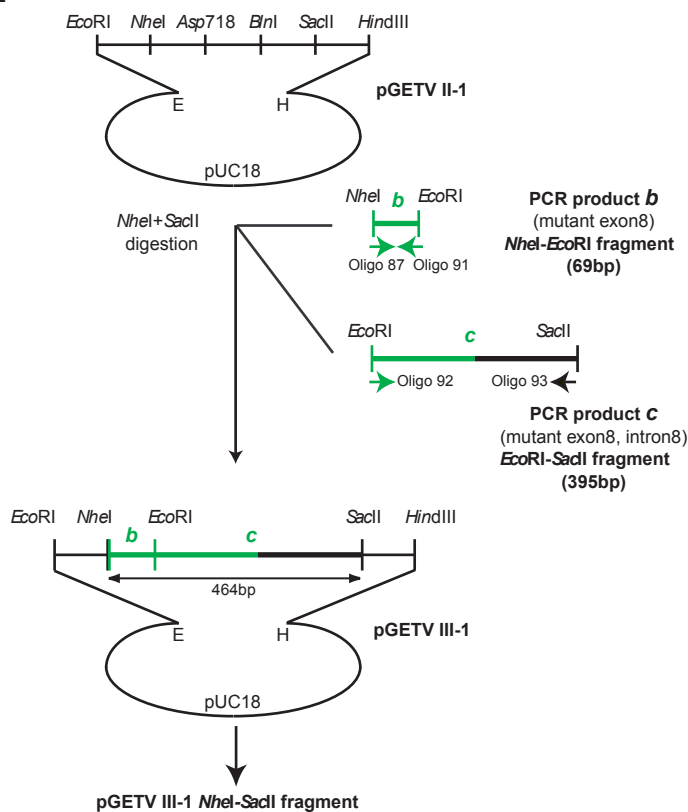

F

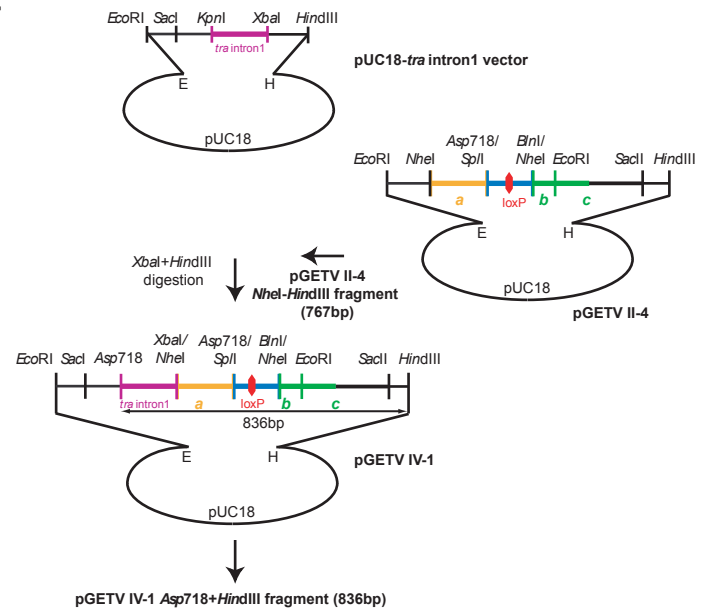

G

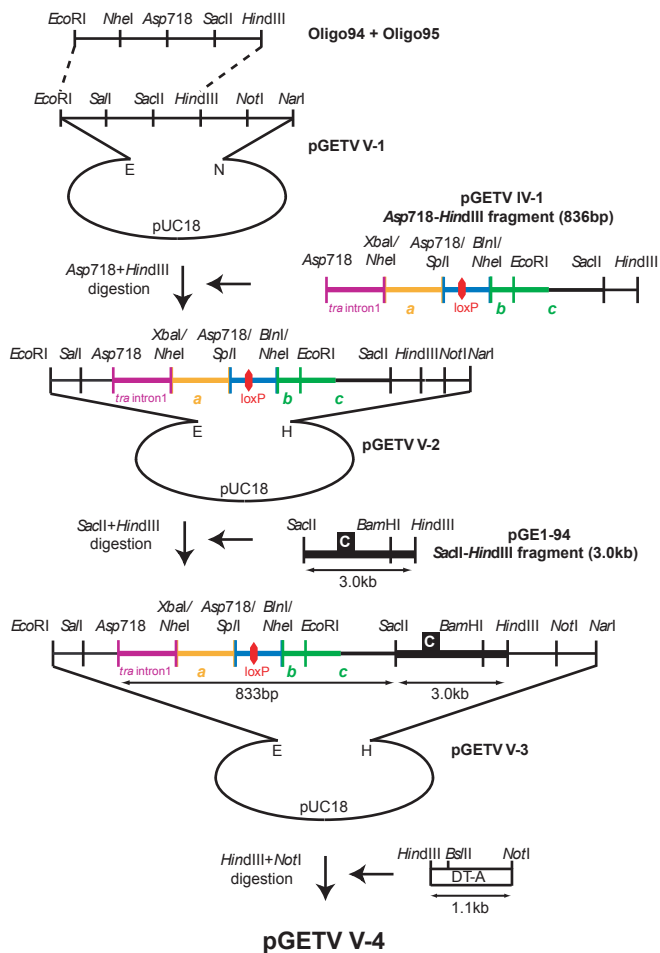

H

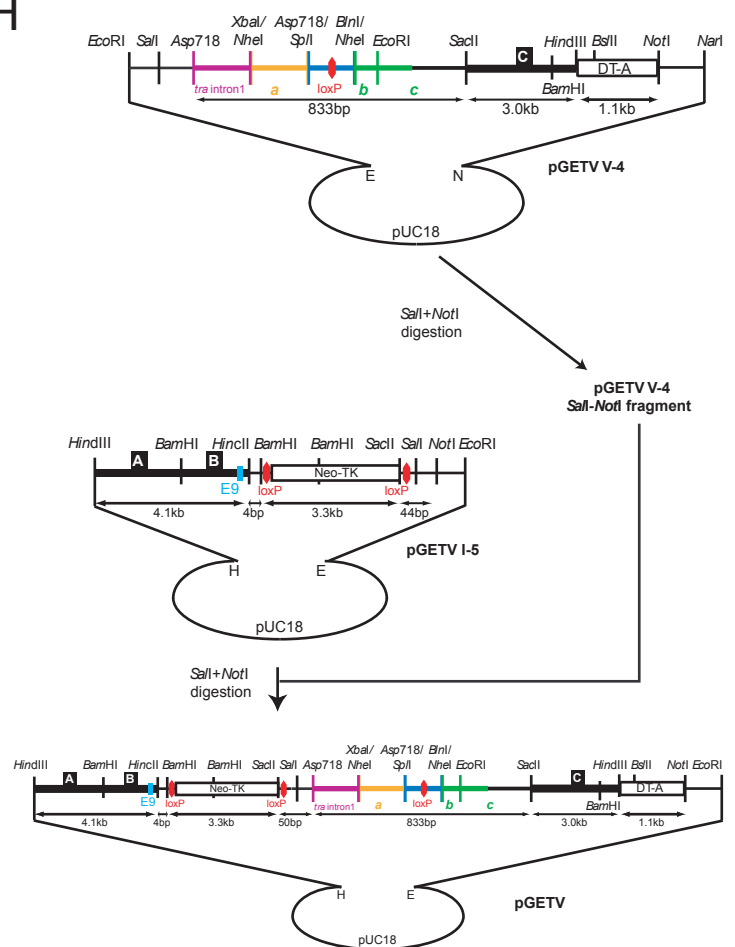

Supplement: Additional file 1: Figure S1 — Genaration of pGETV targeting construct. The loxP sequences (red), the cassette containing the neomycin-resistant and thymidine kinase genes (Neo-TK), the diphtheria toxin A frangment (DT-A) the 5′- and 3′- homologous regions (A, B, and C in bold white letters on black background) of the mouse GluN2A gene, exon9 (light blue), WT exon10 encoding asparagine595 (orange), mutant exon10 encoding glutamine595 (green), the fruit fly tra intron1 (violet), and the mouse β-globin intron (blue) are shown. To clearly illustrate each step of vector construction, the figure shows the relative locations of fragments and relevant restriction sites. The length of each fragment is indicated beneath each fragment. [file 1756-6606-7-44-S1.zip › 1850664515130282_add1/1850664515130282_add2.pdf]
