# Supplementary material for: Enhanced stability of hippocampal place representation caused by reduced magnesium block of NMDA receptors in the dentate gyrus
Source: Mol Brain. 2014 Jun 4;7:44. doi: 10.1186/1756-6606-7-44 (PMC4073519; doi:10.1186/1756-6606-7-44)
Supplement: Additional file 2: Figure S2 — Expression of Cre recombinase in the TDG-Cre mouse. The lacZ expression was visualized using X-gal staining. (A) Whole-brain, (B) cerebellum, (C) thalamus. DG: dentate gyrus, Th: thalamus. Scale bars: 100 μm. [file 1756-6606-7-44-S2.pdf]

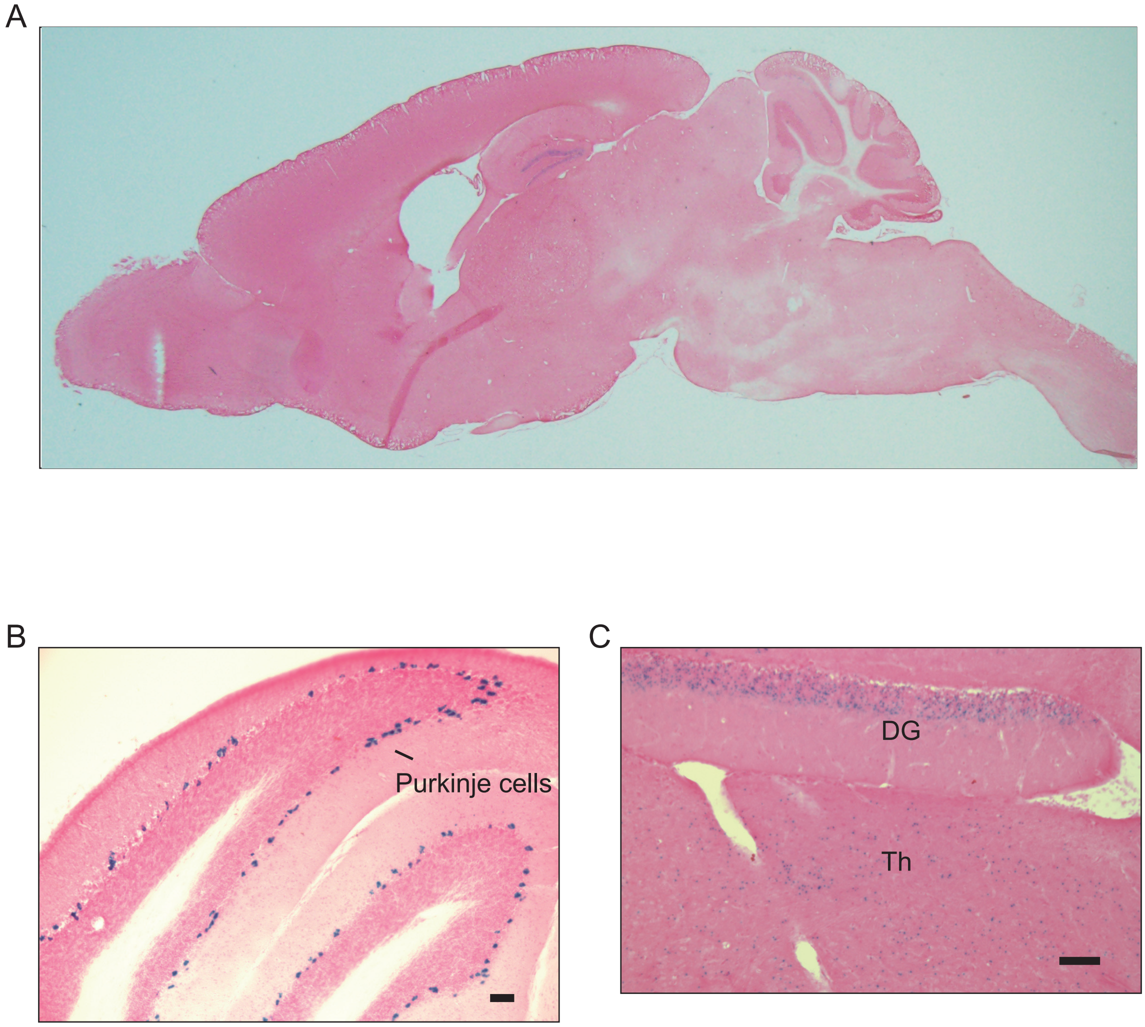

Figure S2, Expression of Cre recombinase in the TDG-Cre mouse.
The lacZ expression was visualized using X-gal staining. (A) Whole-brain, (B) cerebellum, (C) thalamus. DG: dentate gyrus, Th: thalamus. Scale bars: 100 µm.
