# Supplementary material for: Enhanced stability of hippocampal place representation caused by reduced magnesium block of NMDA receptors in the dentate gyrus
Source: Mol Brain. 2014 Jun 4;7:44. doi: 10.1186/1756-6606-7-44 (PMC4073519; doi:10.1186/1756-6606-7-44)
Supplement: Additional file 3: Figure S3 — Rotarod and hot plate tests performance. (A) In the rotarod task, the performance of the mutants was slightly worse than control mice, though this failed to raech significance (day1 (1~3trial): p=0.3247, day2 (4~6trial): p=0.0665). (B) In the hot plate test, there was no difference in the response latency between genotypes. [file 1756-6606-7-44-S3.pdf]

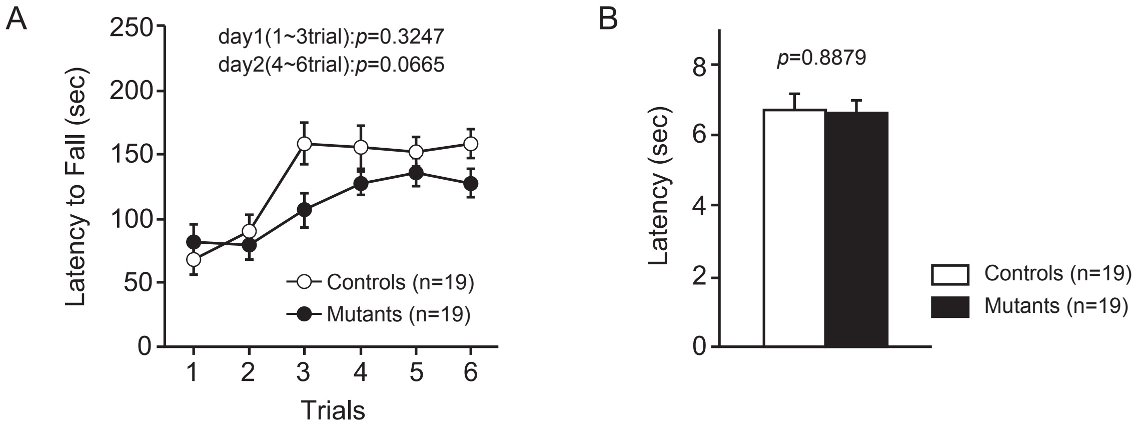

Figure S3, Rotarod and hot plate tests performance.

(A) In the rotarod task, the performance of the mutants was slightly worse than control mice, though this failed to raech significance (day1 (1~3trial): p=0.3247, day2 (4~6trial): p=0.0665).
(B) In the hot plate test, there was no difference in the response latency between genotypes.
