# Supplementary material for: Enhanced stability of hippocampal place representation caused by reduced magnesium block of NMDA receptors in the dentate gyrus
Source: Mol Brain. 2014 Jun 4;7:44. doi: 10.1186/1756-6606-7-44 (PMC4073519; doi:10.1186/1756-6606-7-44)
Supplement: Additional file 4: Figure S4 — Place field maps of pyramidal cells in CA1 region. Place field maps of all CA1 pyramidal cells recorded from control and mutant mice. Maps are sorted by the place field size. Spike waveforms recorded by tetrode were shown on the right side of each place field map. Maximum firing rates were shown at the upper-right of the map (Hz). [file 1756-6606-7-44-S4.pdf]

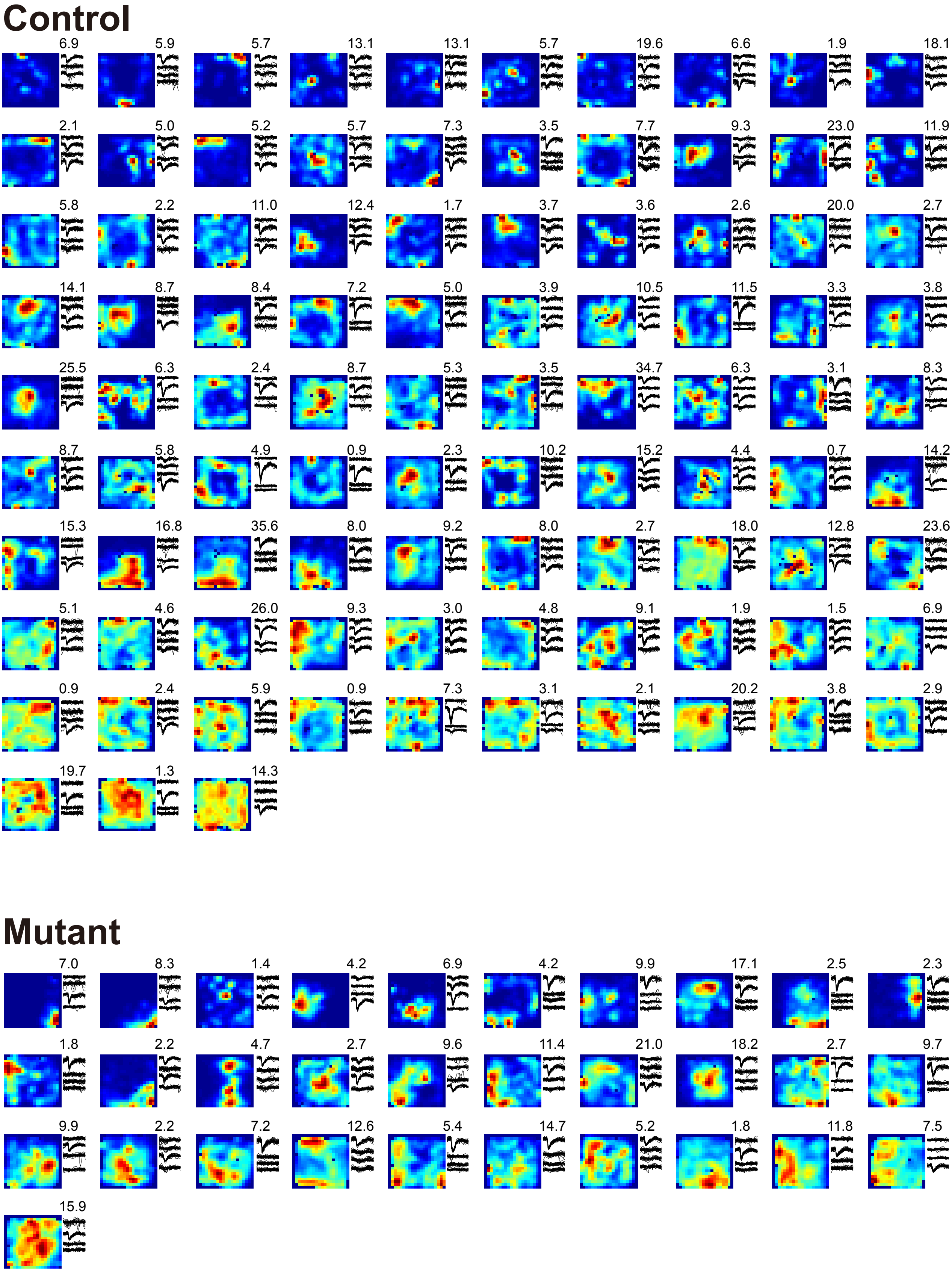

Figure S4, Place field maps of pyramidal cells in CA1 region.
Place field maps of all CA1 pyramidal cells recorded from control and mutant mice. Maps are sorted by the place field size. Spike waveforms recorded by tetrode were shown on the right side of each place field map. Maximum firing rates were shown at the upper-right of the map (Hz).
