# Supplementary material for: Enhanced stability of hippocampal place representation caused by reduced magnesium block of NMDA receptors in the dentate gyrus
Source: Mol Brain. 2014 Jun 4;7:44. doi: 10.1186/1756-6606-7-44 (PMC4073519; doi:10.1186/1756-6606-7-44)
Supplement: Additional file 5: Figure S5 — Modulation of place fields in response to cue rotation. (A) Place field maps of all CA1 pyramidal cells recorded in both original cue environment “ORIG” (left) and rotated cue environment “ROT” (right). Recording time was 15 min each. Maps are sorted by the correlation coefficient between the maps recorded in the two environments. (B) Correlation coefficients between the maps for “ORIG” and “ROT” associated with a 0°, 90°, 180°, or 270° CW rotation of the maps for “ROT”. (C) Correlation coefficients are displayed for two place fields recorded successively in the same environment (1st half of ORIG-2nd half of ORIG) and in cuerotated environment (2nd half of ORIG-1st half of ROT). To quantatevely compare these two conditions, correlation coefficients were calculated for 7.5 min of recordings. Bars indicate mean value and error bars indicate SE. Two-way ANOVA was used for comparisons between groups, followed by a Bonferroni posttest. Asterisks indicate the relative level of the p-value. * p < 0.05, ** p < 0.01. [file 1756-6606-7-44-S5.pdf]

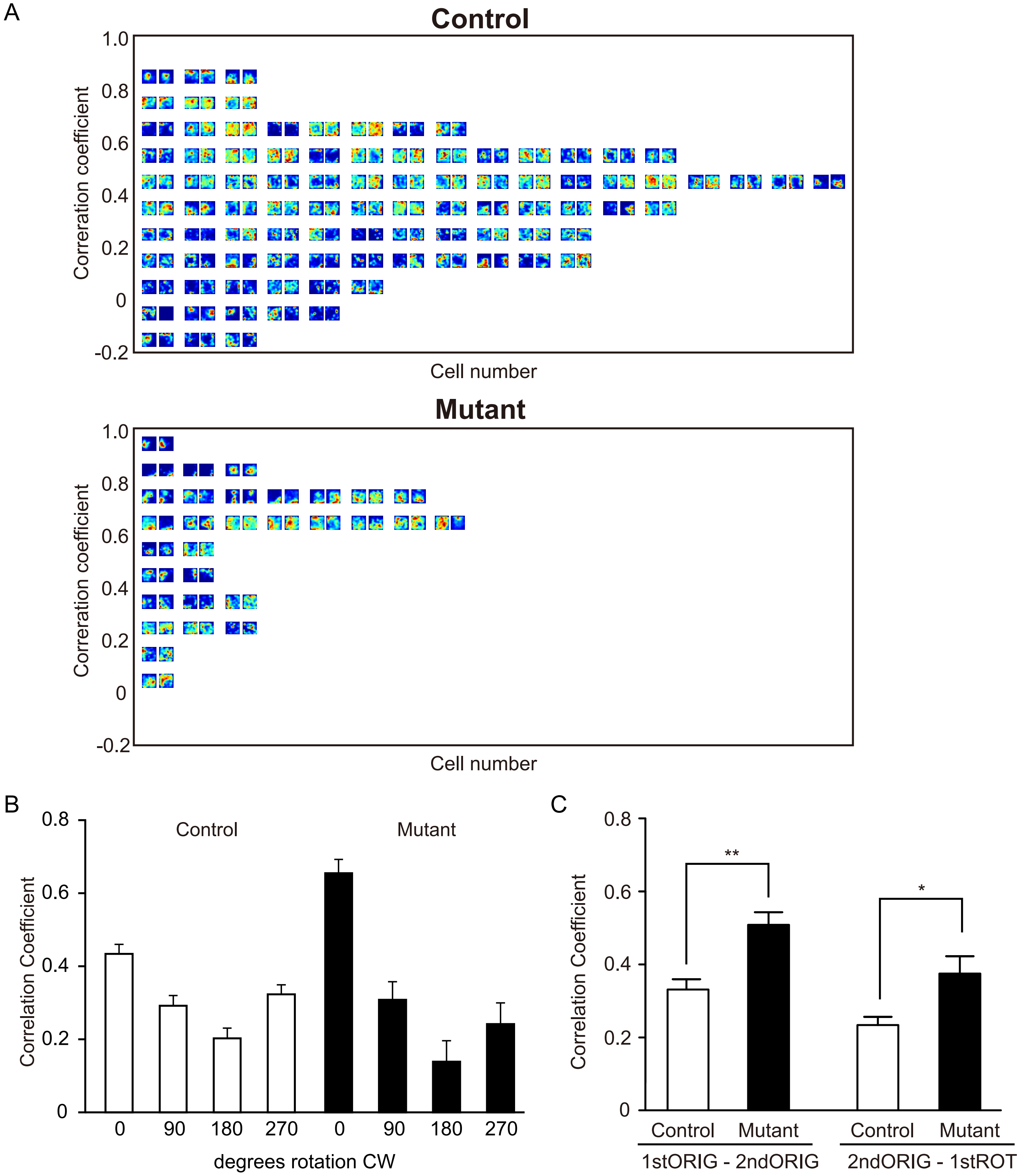

Figure S5, Modulation of place fields in response to cue rotation.
(A) Place field maps of all CA1 pyramidal cells recorded in both original cue environment "ORIG" (left) and rotated cue environment "ROT" (right). Recording time was 15 min each. Maps are sorted by the correlation coefficient between the maps recorded in the two environments. (B) Correlation coefficients between the maps for "ORIG" and "ROT" associated with a 0°, 90°, 180°, or 270° CW rotation of the maps for "ROT". (C) Correlation coefficients are displayed for two place fields recorded successively in the same environment (1st half of ORIG-2nd half of ORIG) and in cue-rotated environment (2nd half of ORIG-1st half of ROT). To quantatevely compare these two conditions, correlation coefficients were calculated for 7.5 min of recordings. Bars indicate mean value and error bars indicate *SE*. Two-way ANOVA was used for comparisons between groups, followed by a Bonferroni posttest. Asterisks indicate the relative level of the p-value. * $p < 0.05$, ** $p < 0.01$.
