# Supplementary material for: Enhanced stability of hippocampal place representation caused by reduced magnesium block of NMDA receptors in the dentate gyrus
Source: Mol Brain. 2014 Jun 4;7:44. doi: 10.1186/1756-6606-7-44 (PMC4073519; doi:10.1186/1756-6606-7-44)
Supplement: Additional file 6: Figure S6 — Place field similarity measurements in distal cue- and local cue-centric coordinate frames. Correlation coefficiens between the two place fields (“original cue” vs “rotated cue”) in control and mutant mice were calculated using distal cue (recording room, door, etc)- and local cue (paper towel)-centric coordinate frames. Data are shown in mean ± SE. Significance was evaluated using the Wilcoxon rank sum test. *p = 0.0016; ** p = 9.0 ×10^-7^. [file 1756-6606-7-44-S6.pdf]

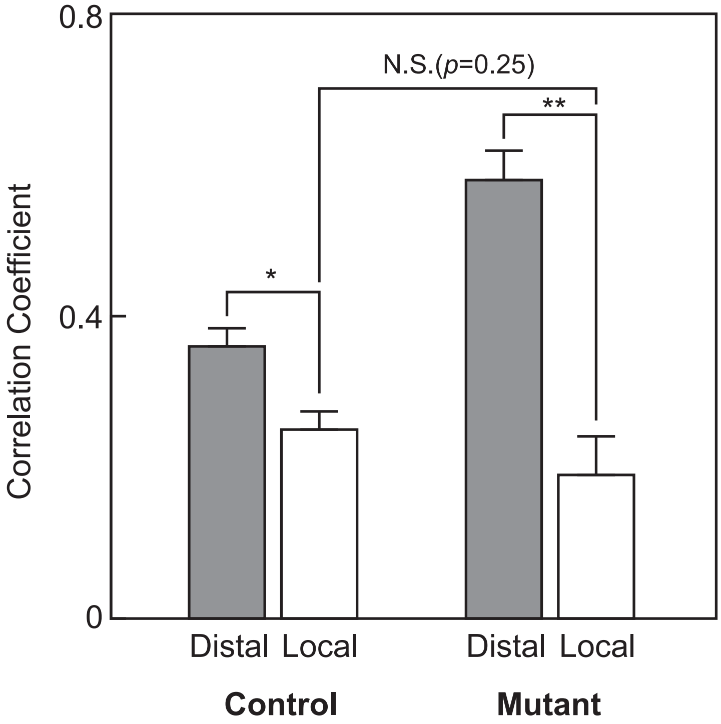

Figure S6, Place field similarity measurements in distal cue- and local cue-centric coordinate frames.

Correlation coefficiens between the two place fields (”original cue” vs “rotated cue”) in control and mutant mice were calculated using distal cue (recording room, door, etc)- and local cue (paper towel)-centric coordinate frames. Data are shown in mean ± SE. Significance was evaluated using the Wilcoxon rank sum test. *$p = 0.0016$; ** $p = 9.0 \times 10^{-7}$.
