# Supplementary material for: Enhanced stability of hippocampal place representation caused by reduced magnesium block of NMDA receptors in the dentate gyrus
Source: Mol Brain. 2014 Jun 4;7:44. doi: 10.1186/1756-6606-7-44 (PMC4073519; doi:10.1186/1756-6606-7-44)
Supplement: Additional file 7: Figure S7 — The number of BrdU-positive cells in the DG. BrdU-positive cells in the subgranular zone of the DG and hilus are not detectably different in the mutant mice and control mice 24 h after BrdU administration. [file 1756-6606-7-44-S7.pdf]

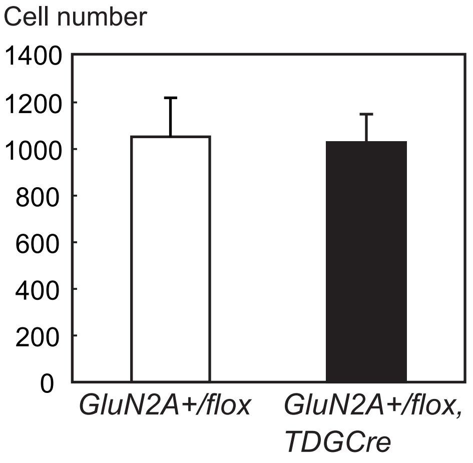

Figure S7, The number of BrdU-positive cells in the DG.
BrdU-positive cells in the subgranular zone of the DG and hilus are not detectably different in the mutant mice and control mice 24 h after BrdU administration.
