# Supplementary material for: Enhanced stability of hippocampal place representation caused by reduced magnesium block of NMDA receptors in the dentate gyrus
Source: Mol Brain. 2014 Jun 4;7:44. doi: 10.1186/1756-6606-7-44 (PMC4073519; doi:10.1186/1756-6606-7-44)
Supplement: Additional file 8: Figure S8 — NMDA receptors formed with the wild type GluN2A (N) or the mutant GluN2A(Q) subunit. In Cre-expressing cells of GluN2A+/flox mice, NMDA receptors will consist of the three populations (middle column). Left column and right column shows the GluN2A+/flox and GluN2Aflox/flox-TDGCre mice respectively. [file 1756-6606-7-44-S8.pdf]

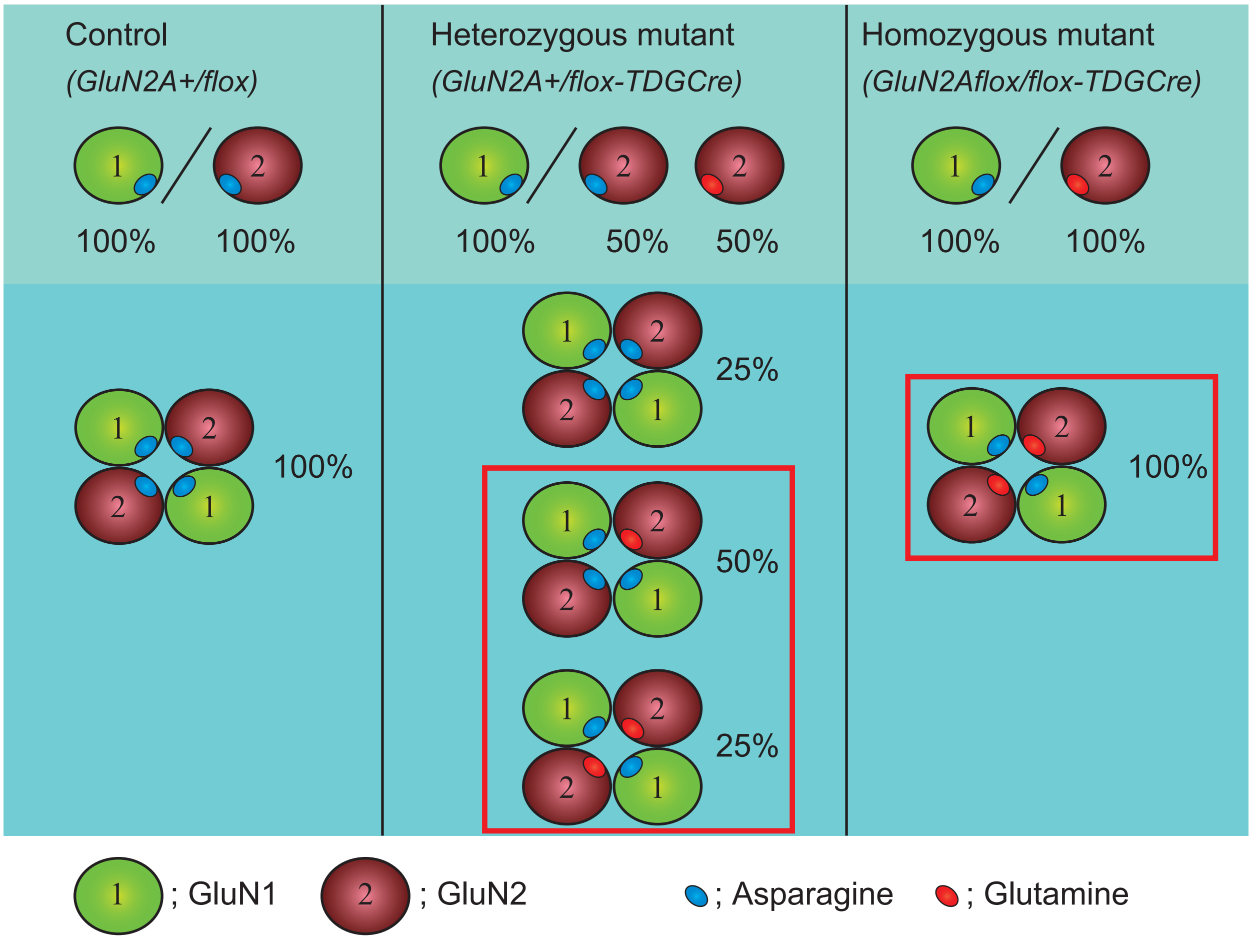

Fig.S8 NMDA receptors formed with the wild type GluN2A (N) or the mutant GluN2A(Q) subunit. In Cre-expressing cells of *GluN2A+/flox* mice, NMDA receptors will consist of the three populations (middle column). Left column and right column shows the *GluN2A+/flox* and *GluN2Aflox/flox-TDGCre* mice respectively.
